# Specialized metabolites from plants as a source of new multi-target antiviral drugs: a systematic review

**DOI:** 10.1007/s11101-023-09855-2

**Published:** 2023-03-12

**Authors:** Maria Ponticelli, Maria Laura Bellone, Valentina Parisi, Annamaria Iannuzzi, Alessandra Braca, Nunziatina de Tommasi, Daniela Russo, Annalisa Sileo, Paola Quaranta, Giulia Freer, Mauro Pistello, Luigi Milella

**Affiliations:** 1 Department of Science, University of Basilicata, Viale Dell’ateneo Lucano 10, 85100 Potenza, Italy; 2 Department of Pharmacy, University of Salerno, Via Giovanni Paolo II 132, 84084 Fisciano, Salerno, Italy; 3 Ph.D. Program in Drug Discovery and Development, Department of Pharmacy, University of Salerno, Via Giovanni Paolo II 132, 84084 Fisciano, Salerno, Italy; 4 Department of Pharmacy, University of Pisa, Via Bonanno 33, 56126 Pisa, Italy; 5 Interdepartmental Research Center “Nutraceuticals and Food for Health”, University of Pisa, 56100 Pisa, Italy; 6 Retrovirus Center, Virology Section, Department of Translational Research and New Technologies in Medicine and Surgery, University of Pisa, Pisa, Italy; 7 Virology Unit, Pisa University Hospital, Pisa, Italy

**Keywords:** Natural compounds, Antiviral activity, In vivo studies, Antivirals mechanism of actions, Systematic review

## Abstract

Viral infections have always been the main global health challenge, as several potentially lethal viruses, including the hepatitis virus, herpes virus, and influenza virus, have affected human health for decades. Unfortunately, most licensed antiviral drugs are characterized by many adverse reactions and, in the long-term therapy, also develop viral resistance; for these reasons, researchers have focused their attention on investigating potential antiviral molecules from plants. Natural resources indeed offer a variety of specialized therapeutic metabolites that have been demonstrated to inhibit viral entry into the host cells and replication through the regulation of viral absorption, cell receptor binding, and competition for the activation of intracellular signaling pathways. Many active phytochemicals, including flavonoids, lignans, terpenoids, coumarins, saponins, alkaloids, etc., have been identified as potential candidates for preventing and treating viral infections. Using a systematic approach, this review summarises the knowledge obtained to date on the in vivo antiviral activity of specialized metabolites extracted from plant matrices by focusing on their mechanism of action.

## Introduction

Viruses are ubiquitous organisms that depend on host structures to replicate; they exist in all environments and may infect a broad spectrum of life forms, from plants to bacteria and animals. Structurally viruses are formed by two essential elements: the nucleic acid genome, consisting of single-stranded or double- RNA or DNA, and a capsid that packs and protects the viral genome and plays a role in the host cell viral entry. Apart from capsid, some viruses possess an additional protective layer known as the envelope, which may be formed by lipids or glycoprotein (Cassedy et al. 2021). Based on the presence of the envelope, it is possible to distinguish enveloped viruses like herpes virus simplex 1 and 2 (HSV-1, HSV-2), cytomegalovirus (HCMV), respiratory syncytial virus (RSV), and influenza A virus (IAV), and non enveloped viruses such as coxsackievirus B4 (CVB3), rotavirus (RV), and enterovirus 71 (EV71). The diffusion of viral infections is responsible for pandemics development. Over the course of history, there have been several outbreaks of disease caused by viral infections, among them the Spanish flu pandemic (1918–1920), smallpox (1972), HIV epidemic (1981), SARS (2003), H1N1 pandemic (2009), Ebola Virus (2014–2016), Zika Virus (2015–2016), until the pneumonia cases of December 2019 baptized by the WHO with the name of novel coronavirus (SARS-CoV-2). The issue of a pandemic is a global problem, and the development of efficient antivirals is the only way to accelerate the return to normal conditions. However, one of the virus infection problems is the occurrence of resistance to the generally used drugs. Viruses are indeed known to rapidly mutate their genome during successive replications, determining the chance for increased antiviral drug resistance as was seen for human viral diseases like hepatitis B, hepatitis C, herpes simplex virus, and influenza virus (Kumar et al. 2020). The modern approach to antiviral drug discovery is to study the viral structure and replication details to find targets for new antiviral drugs (Malone et al. 2022; Shaker et al. 2021). Along with designing tailormade drugs against specific viral proteins of defined species, a more traditional strategy to increase the number of drugs available to treat viral diseases is to screen natural compounds derived from plants. In line with the traditional Chinese medicine theory, “*Everything has its own enemy from nature*”, meaning that in the world, everything has its proper method of survival and its way of destruction, thereby preserving nature’s balance. In fact, it is possible to obtain from nature the constituents for good health if correctly used (Yao et al. 2009). Seeking drugs from medicinal plants has been a practice since ancient times, but it can now benefit from the use of the continuously growing number of techniques that scientific progress can offer. Natural products are an important source of numerous therapeutic compounds exhibiting antiviral properties against several viruses. Whether the antiviral molecules are synthetic or natural, a distinction can be made between direct-acting and indirect-acting antivirals. Direct antivirals block viral proteins and enzymes or inhibit viral pathways essential for viral replication, thus acting against one or more phases of viral replication. On the other hand, indirect antivirals interfere with host intracellular pathways that viruses exploit and hijack to their advantage without interfering with the normal function of non-infected cells so as to be effective and safe (Lou et al. 2014). Noteworthy is also the antiviral effect exerted by regulating the host immune system and host levels of radical oxygen species. Several isolated bioactive molecules of natural origins, such as terpenes, flavonoids, coumarins, alkaloids, lignans, and others, have been reported to possess interesting antiviral properties by acting with multiple mechanisms of action. Through in vitro and in vivo investigations, it was indeed demonstrated that most of these natural compounds might directly inhibit viral infection and replication stages and/or regulate host intracellular signaling pathways, which are essential for virus survival, and, thus, the host immune state (Mukhtar et al. 2008; Brindisi et al. 2020). This review illustrates some natural compounds active in vivo against human viruses, such as influenza viruses, arbovirus, herpetic viruses, retroviruses, hepatitis viruses, enteroviruses, and coronaviruses.

## Methods

### Search strategy

This systematic review was conducted through a literature search carried out in April–May 2022 including all results published up to date. For the literature search, three database (Pubmed, Scopus, and SciFinder) were questioned using the combination of the three keywords: “antiviral” and “benzoic acid” or “anthocyanidin” or “aurone” or “catechin” or “chalcone” or “cinnamic acid” or “cyanidin” or “depside” or “ depsidone” or “flavanone” or “flavone” or “flavonoid” or “flavonols” or “isoflavan” or “isoflavone” or “phenylpropanoid” or “stilbenoid” or “tannin” or “terpen” or “monoterpene” or “sesquiterpene” or “diterpene” or “triterpene” or “sesterterpene” or “tetraterpene” or “carotenoid” or “saponin” or “alkaloid” or “coumarin” or “diarylheptanoid” or “stirilpiron” or “quinone” or “lignan” or “flavolignan” or “coumestan” and “in vivo” or “clinical” or “clinical trial” or “preclinical” or “mice” or “rat” or “animal” or “patient”. Additional research was conducted by using CoVid-19 or SARS-CoV-2 or coronavirus as keywords.

Investigators were not contacted and unpublished data were not considered. The review was performed according to the Preferred Reporting Items for Systematic Reviews and Meta-Analyses (PRISMA) statement (Faraone et al. 2020a; Porreca et al. 2021).

### Study selection

Two investigators (LM and AB) selected the manuscripts by screening titles, abstracts, and finally full texts. In cases of disagreements, a third reviewer (NDT) was consulted.

The screening phase of the manuscript was based on the exclusion criteria for the title and abstract as: not in vivo study (clinical and preclinical), not natural compound (semisynthetic or synthetic derivatives were excluded), no single compound (extracts, fractions, enriched extracts were not included), not disease of interest (antiviral). Also patent, review articles, meta-analysis, abstracts, conferences, editorials/letters, case reports, and conference proceedings were excluded from this systematic review. Additional exclusion criteria were used for full-text screening: full-text not available, language (only articles published in English were analysed), double publication. Pure compounds extracted from natural matrixes and pure commercial compounds were included. The selected articles were carefully reviewed to identify and exclude the reports that did not fit the criteria described above. During the analysis of the references in the selected manuscripts, additional research was carried out to include other studies that do not meet the selected keywords.

### Data extraction

Each selected full text has been examined by the authors, and data were collected. In addition, the following information were recorded and tabulated: compound, source, virus type, experimental model, dose, mechanism of action, and outcome measure.

### Methodological quality assessment

The risk of bias and quality of the in vivo investigations was assessed (Faraone et al. 2020b) to evaluate the study's quality, including the randomization of the treatment allocation, blinded drug administration, blinded outcome assessment, and outcome measurements. Studies that report information regarding randomization of animals, blinding, and outcome measurements have higher methodological quality.

## Results and Discussion

### Search outcomes

**Fig. 1:**
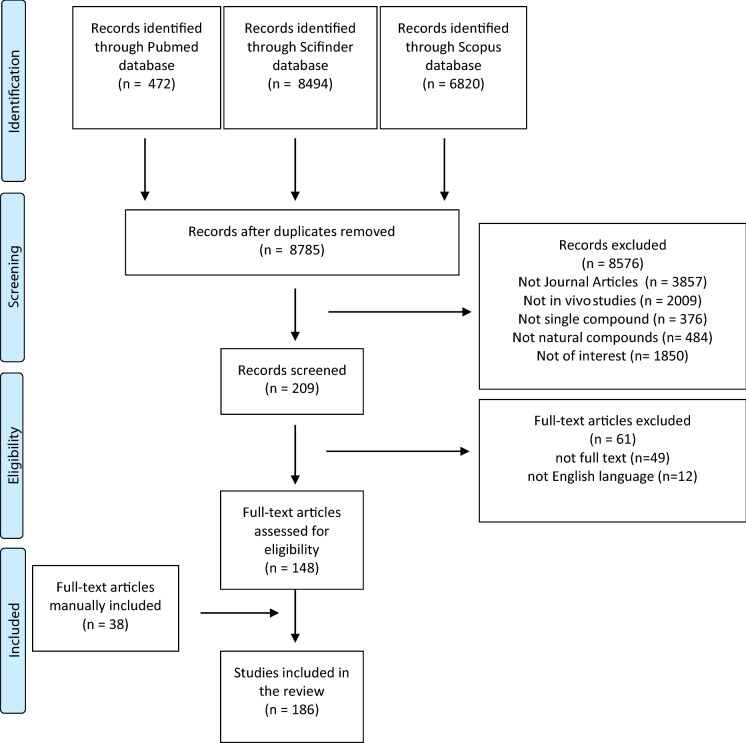
PRISMA flow chart of the systematic review on natural antivirals

The primary search identified 15,786 reports (472 from PubMed, 8494 from SciFinder, and 6820 from Scopus). However, 7001 manuscripts were indexed in more databases and were considered only once, resulting in 8785 original articles. After an initial screening of titles and abstracts, 8576 articles were excluded since they did not meet the inclusion criteria. Finally, 209 articles were fully analyzed, and among these, 61 studies were excluded for the language and the not available full text, but other 38 reports were added after manual research searching by single molecules. In total, 186 articles were included for data extraction. A flowchart describing the progressive study selection and numbers at each stage is shown in Fig. 1. The articles selected for this review were categorically analyzed in relation to the year of publication, the country where the study was conducted, and the natural compounds evaluated as antiviral agents on in vivo studies.

**Fig. 2:**
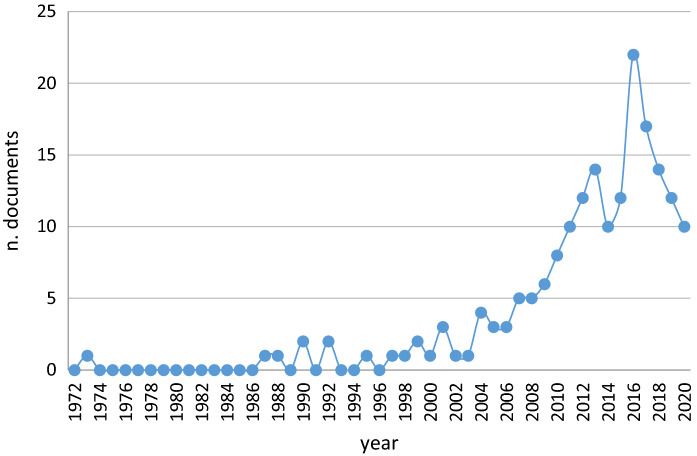
Number of documents per year on natural antiviral agents

**Fig. 3:**
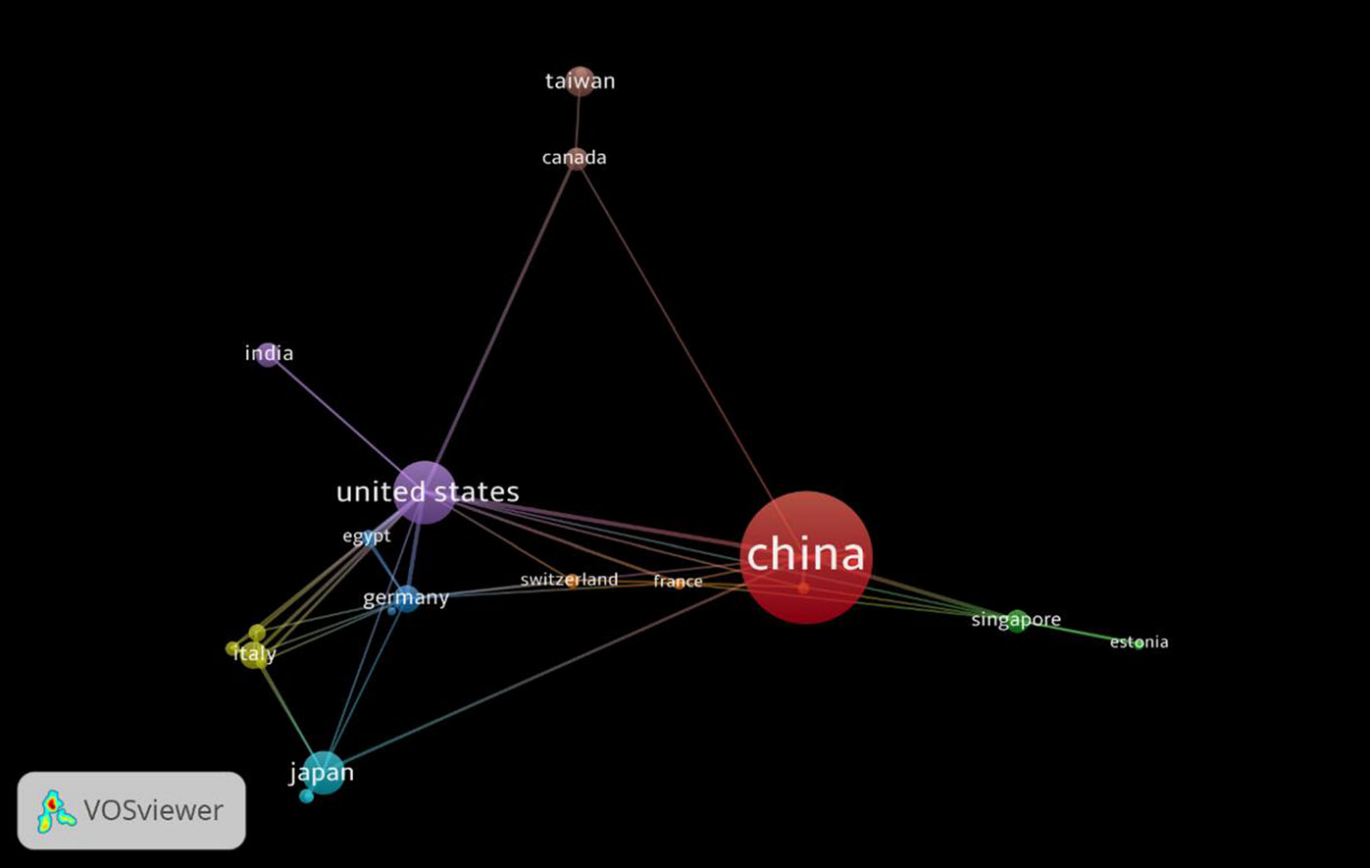
VOSviewer network visualization map of country co-authorship (International collaboration). Thirty-two countries had at least 1 publication; the largest set of connected countries consists of 28 countries in 8 clusters. Different colour refers to the cluster to which an item belongs, lines represent links between items, while the larger the item circle, the higher the item weight

**Fig. 4:**
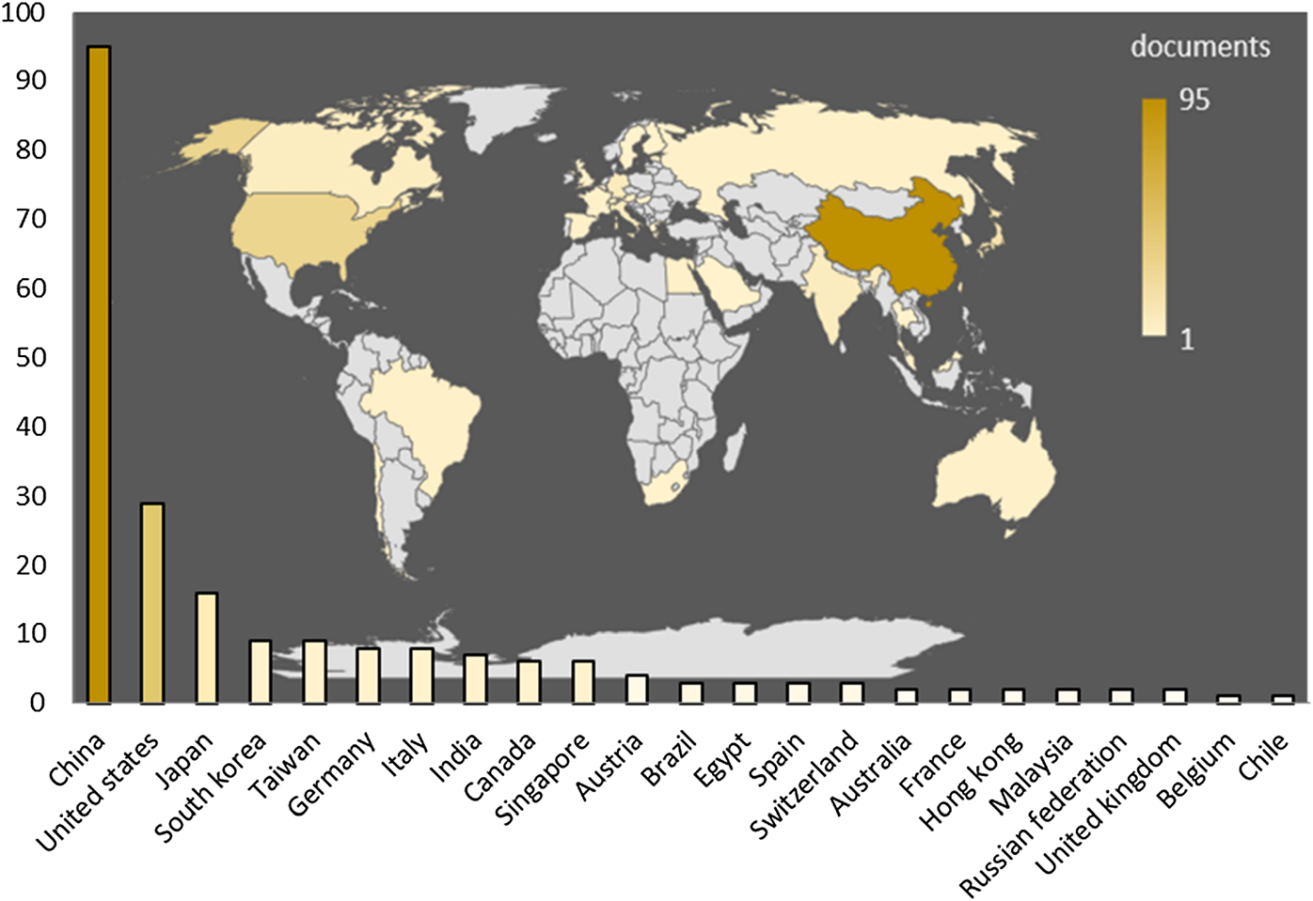
Numbers of documents per country included into the systematic review

The first report included in this review is dated 1968, but the largest number of publications was published from 2010 with an increasing trend (141 papers, 76%) with at least 10 reports per year (Fig. 2). Co-authorship research is an important bibliometric factor and the level of research collaboration is an index to assess the current status of research in a specific field. Country co-authorship analysis is an important form of co-authorship analysis by reflects the degree of communication between countries as well as the influential countries in this field. Based on the bibliographic data collected, the countries’ co-authorship network visualization map was created with VOSviewer (Fig. 3). In the process of mapping the minimum document threshold of a country was set at 1; there were 4 countries out of 32 listed as visualization items. The big nodes represent the influential countries, whereas the links between nodes represent the cooperative relationships among institutes. The distance between the nodes and the thickness of the links represent the level of cooperation among countries. China was at the centre of the research, and the main partners were USA, Japan and Germany. Considering the number of documents, the country lead in the research of natural compounds as antiviral agents by in vivo studies was China with 95 documents (41.1%), followed by United States of America (29 documents, 12.6%), Japan (16 documents, 6.9%), South Korea, and Taiwan (9 documents, 3.9%) (Fig. 4).

**Fig. 5:**
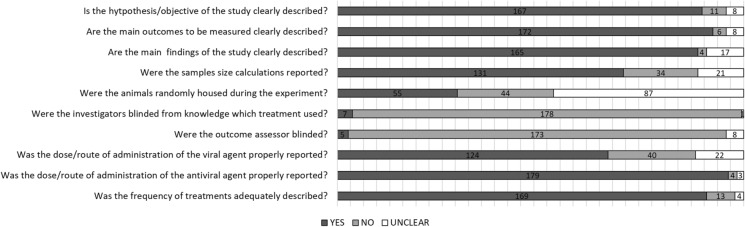
Methodological quality assessment of included studies. Dark gray bars indicate the studies that met each criterion; light gray bars indicate the studies that did not and white bars indicate the studies with unclear answers

Concerning quality assessment, all in vivo studies were carefully analysed through a standard checklist. As reported in Fig. 5, almost all studies described the objectives, outcomes to be measured, main findings obtained as well as the route of administration and the frequency of treatments. In addition, 55 studies (29.6%) established that the allocation was randomized, and 70.4% of the included articles reported sample size calculations.

### Phytochemicals and antiviral activity

The World Health Organisation (WHO) encourages the use of herbal medicines as remedies to overcome the absence or inactivity of conventional therapy. Emphasis is placed on the study of specialized metabolites extracted from natural sources, their chemical structure and pharmacological activity in order to use them for treating or preventing illnesses with lower toxicity than the existing molecules. In this context phytochemicals, such as flavonoids, terpenoids, coumarins, lignans, saponins, etc., are reported to regulate viral cellular functions, permeability throughout the host membrane, and replication, making them potential molecules for producing new effective anti-viral drugs. Table 1 listed the main natural active molecules with proved in vivo anti-viral activity, while in the text, their mechanism of action was treated.

**Table 1:** Plant molecules with proven in vivo antiviral activity

Compound	Source	Virus type	Animal model and dose	Mechanism of action	Results	References
*Terpenes*						
Monoterpenes						
	*Eucalyptus* genus	IAV H1N1, FM1	Female BALB/c mice, 30, 60, and 120 mg/kg	Inhibition of inflammatory response and recruitment of early lymphocytes	↑ Survival rate ↓ mRNA Expression of influenza virus M gene ↓ IL-1β, IL-6, TNF-α, IFN-γ in lung tissue ↓ IL-4, IL-5, IL-10, MCP-1 in nasal lavage fluids ↓ ICAM-1, VCAM-1, and NF-κB p65 in lung tissue	Li et al. (2016)
		IAV (H3N2)	BALB/c mice, 30, 60, and 120 mg/kg/day (plus 0.1, 0.2, 0.4 mg/kg oseltamivir)	Not specified	↑ Survival rate ↓ IL-1β, IL-10, TNF-α, IFN-γ levels in lung tissue	Lai et al. (2017)
	*Paeonia lactiflora* Pall. roots	Influenza virus A/ FM/1/47	Mice, 50 and 100 mg/kg	Down-regulation of αv*β*3/TGF-*β*1 pathway in lung tissue	↓ Pulmonary fibrotic markers ↓ Col I ↓ Col III ↓ αvβ3, TGF-*β*1, Smad2, NF-*κ*B, and p38MAPK expression	Yu et al. (2021)
	*Mosla chinensis* Maxim	Influenza virus (A/H1N1)	C57BL/6 mice, 50 mg/kg/day	Regulation of the TLR/RLR pattern recognition and related CD4 + T cells	↓ Pro-inflammatory cytokines ↓ Expression of RIG-I, MyD88 and NF-κB proteins	Zheng et al. (2021)
*Iridoids*						
	*Nyctanthes arbor-tristis* L	Semliki forest virus	CDRI mice, 0.5 mg/mouse	Not specified	↑ Survival rate ↑ Protection (%)	Rathore et al. (1990)
	*Nyctanthes arbor-tristis* L	Semliki forest virus	CDRI mice, 0.5 mg/mouse	Not specified	↑ Survival rate ↑ Protection (%)	Rathore et al. (1990)
	*Gardenia jasminoides* J. Ellis	A/Jiangsu/1/2009 (H1N1) Influenza virus	Female ICR mice, 5, 10, and 20 mg/kg	Unclear	↑ Survival rate ↓ Clinical signs ↓ Body weight loss ↓ Viral replication ↓ Severity of viral lung lesion ↓ IL-6, TNF-α, IFN-γ levels in lung tissue ↑ IL-4 and IL-10 release in lung tissue	Zhang et al. (2017b)
	*Jasminum officinale* var. *grandiflorum* L	HBV	Ducklings, 80 mg/kg	Unclear, supposed alteration in HBsAg gene transcription	↓ Viral DNA level in serum (80 mg/kg)	Zhao et al. (2009a)
*Sesquiterpenes*						
	*Atractylodes macrocephala* Koidz	IAV A/PR/8/34 (H1N1) A/shenzhen/203/2001 (H3N2)	Male ICR mi, 10 and 40 mg/kg	Block of virus adsorption to host cells or inhibition of virus replication	↓ Lung index ↓ Histological damage ↓ Virus load ↑ IFN-β serum level ↓ IL-6, TNF-α and IL-1β in serum	Cheng et al. (2016)
	*Centipeda minima* (L.) A. Braun&Asch	IAV A/PR/8/34 (H1N1)	Female BALB/c mice, 10 and 25 mg/kd/day	Role in viral RNA synthesis and protein expression	↑ Survival rate (25 mg/kg) ↓ Body weight loss (25 mg/kg)	Zhang et al. (2019)
	*Curcuma* genus	H1N1 virus	Specific pathogen–free BALB/c mice, 100 mg/kg	Not specified	↓ Viral replication	Li et al. (2020a)
	*Curcuma longa* L	IAV A/PR8/34 (H1N1)	BALB/c mice, 50, 100 mg/kg/day and 100 mg/kg/day plus 1 mg/kg/day oseltamivir	↓ Attachment/entry to host cells ↓ Viral protein expression ↓ Viral RNA replication	↑ Survival rate ↑ Delay in mortality ↓ Virus load	Liao et al. (2013)
		H1N1 IAV	Specific pathogen–free BALB/c mice, 100 mg/kg	Not specified	↓ Virus load ↓ Lung index protective effect on mice at the early stage of H1N1 infection ↓ Viral replication ↓ Histological damage ↓ TAP1 expression	Li et al. (2020a)
	*Pogostemon cablin* (Blanco) Benth	IAV A/Leningrad/134/17/1957 H2N2)	Kunming mice, 1 and 5 mg/kg/day	Interference with neuraminidase function	↑ Survival rate	Wu et al. (2011a)
		IAV (A/FM/1/47 H1N1, FM1)	Sparse-fur mice, 20, 40, and 80 mg/kg/day	Not specified	↑ Survival rate ↓ mRNA Expression of influenza virus M gene ↑ Serum anti-IAV IgA level (40–80 mg/kg/day) ↑ Lung anti-IAV IgG level (40–80 mg/kg/day) ↑ Blood CD3 + and CD4 + T cell percentages ↓ Blood CD8 + cell levels ↑ IL-10 production in serum (80 mg/kg/day) ↓ TNF-α production in serum and lung (40–80 mg/kg/day) ↑ IFN-γ and IL-10 production ↓ Lung inflammation ↓ Lung histopathology score	Li et al. (2012)
		IAV H1N1 (A/PR/8/34)	Female Kunming mice, 20–40 µg/day	Block viral infection through targeting virus particles and cellular PI3K/Akt and ERK/MAPK signaling pathways	↓ Pulmonary viral titers ↑ Survival rate (40 µg/day) ↓ Pneumonia symptoms ↓ Pulmonary histological damage	Yu et al. (2019)
	*Stachybotrys* genus	IAV A/Kumamoto/5/67(H2N2)	Male BALB/c mice, 20 and 10 mg/mouse	Not specified	↓ Pulmonary virus load (10 mg/mouse PEG 400)	(Yagi et al. 1999)
		A/WSN/1933 (H1N1)	Female BALB/c mice, 100 μL of solutions containing various concentrations (4, 20, 100 mg/kg/day) of stachyflin in PEG 400	Not specified	↓ Virus titers in lung (100 mg/kg/day)	Motohashi et al. (2013)
		A/chicken/Ibaraki/1/2005 (H5N2)				
*Diterpenes*						
	*Andrographis paniculata* (Burm*.*f.) Nees	IAV A/duck/Hubei/XN/2007 (H5N1)	BALB/c female mice, 500 and 1000 mg/kg/day	↓ Release of the proinflammatory cytokines/chemokines	↑ Survival rate ↓ Body weight loss ↓ Lung pathology ↓ TNF-α, IL-1β, IL-6, CCL-2 and CXCL-10, INF- α, INF- β, INF-γ, MIP-1 α, MIP-1 β in serum	Cai et al. (2016)
		IAV A/chicken/Hubei/327/2004 (H5N1)			↑ Survival rate ↓ Body weight loss ↓ Lung pathology ↓ TNF- α, IL-1 β, IL-6, CCL-, CXCL-10 in serum	
		A/PR/8/34 (H1N1)			↑ Survival rate ↓ Body weight loss ↓ Lung pathology ↓ TNF- α, IL-1β, IL-6, CCL-2 and CXCL-10 in serum	
	*Andrographis paniculata* (Burm*.*f.) Nees	HIV	Human patients, 5 mg/kg body weight (three times a day), 10 mg/kg body weight (three times a day)	Inhibition virally induced dysregulation of cell signaling pathways in infected cells and inhibition of decline of the CD4 + T cell population	Very modest ↓ of virus load at 10 mg/kg ↑ Blood CD4 + lymphocyte counts	Calabrese et al. (2000)
		IAV A/Chicken/Guangdong/ 96 (H9N2)	Female BALB/c mice, 125, 250, 500, 1000, and 2000 mg/kg/day	Not specified	↑ Survival rate (500 mg/kg/day) ↓ Virus titer in lung tissue (500 mg/kg/day)	Chen et al. (2009)
		A/Duck/Guangdong/99 (H5N1)				
		IAV H1N1				
		DENV (type 2 strain 16,681)	ICR suckling mice, 10 mg/kg intraperitoneal	↑ Heme oxygenase 1 expression	↑ Survival rate ↑ Body weight ↑ Life-span ↓ Virus titer	Tseng et al. (2016)
		IAV A/PR/8/34 (H1N1)/	Female C57BL/6 (C57) mice, 10 mg/kg	↓ Expression of JAK/STAT signals	↑ Survival rate (both in simultaneous and in delayed treatment) ↑ Body weight	Ding et al. (2017)
	*Cephalosporium aphidicola* Petch	HSV-1	Dutch female rabbits 1, 10 mg/mL applied as aqueous drops hourly for 7 h	Inhibition of DNA synthesis	↓ Maximal lesion score (10 mg/mL)	Bucknall et al. (1973)
			Guinea pigs, 100 mg/kg	Not specified	Not observed	
	*Scoparia dulcis* L	HSV-1	Female golden hamster, 20, 100, and 200 mg/kg/day	Role in early stages of viral infection (fusion of the viral envelope with plasmatic membrane, transport of capsids through nuclear pores and release of the DNA into the nucleus)	↓ Corneal lesions ↑ Survival time	Hayashi et al. (1988)
*Sesterterpenes*						
	*Bipolaris oryzae* (Breda de Haan) Shoemaker	IAV	Balb/c mice, 0.3 mg/kg/mouse	Not specified	↓ Symptom severity (pulmonary lesions, inflammation, atrophy of thymus and spleen) ↓ Viral titers in lungs ↓ Hemagglutinin ↓ Nucleoprotein ↑ IL-28 ↑ ISG15 ↑ ISG20	Wang et al. (2016)
*Triterpenes*						
	*Panax notoginseng* (Burk.) F.H. Chen	CVB3	Male BALB/c mice, 100, 200, and 400 mg/kg/day	Not specified	↓ Virus titers in the hearts (200, 400 mg/kg/day) ↓ Myocardium damage ↓ Mononuclear cell infiltration ↑ Lactate dehydrogenase level in plasma ↑ Creatine kinase level in plasma	Wang et al. (2012)
	*Anemone chinensis* Bunge	HBV	Male HBV-transgenic mice, 2 mg/kg	Specific suppression of SOD2 expression	↓ HBsAg and HBeAg serum levels ↓ HBV DNA replication ↓ SOD2 mRNA expression in liver ↓ Protein levels and activity of SOD2 in liver ↓ Protein level of pCREB in liver ↑ Caspase 3 activity in liver ↑ Superoxide anion generation in liver tissue	Yao et al. (2009)
	*Ziziphus jujuba* Mill	IAV A/PR/8/34 (H1N1) virus	C57BL/6 mice, 10 mg/kg/dose	Not specified	↓ Pulmonary pathology ↓ INF-γ levels in lung	Hong et al. (2015)
	*Tripterygium wilfordii* Hook. f	DENV type 2 (strain 166,681-PDK53) DENV-2	ICR suckling mice, 0.1 mg/kg	Inhibitory effect on DENV through upregulation of IFN expression and activation of downstream JAK-STAT signaling	↑ Survival rate ↑ IFN-α-2, IFN-α-5, OAS1, OAS2, and OAS3 gene expression levels in brain tissue	Yu et al. (2017a)
	*Carissa spinarum* L	7401H HSV-1	BALB/C mice, 5, 10 and, 20 mg/kg/day	Not specified	↓ Progression of infection (20 mg/kg) ↑ Mean survival times	Tolo et al. (2010)
	*Helicteres angustifolia* L	HBV	Pekin ducklings, 50, 100 mg/kg/day	Not specified	↓ Virus replication levels in serum (50–100 mg/kg) ↓ Relaxed circular, linear, and single-stranded forms of virus DNA in the liver ↓ cccDNA in liver tissue ↓ Histopathological damage (100 mg/kg)	Huang et al. (2013a)
	*Schisandra sphenanthera* Rehd. et Wils	HCV	Transgenic ICR^R+^ mice, 5 mg/kg/day	Block HCV entry by inhibiting the fusion process due to alteration host membrane’s fluidity	↓ HCV RNA levels in serum ↓ Number of HCV NS3- or core protein-positive hepatocytes in liver samples	Qian et al. (2016)
	Often found in plant kingdom	HSV-1	Female C57BL/6 mice treated with gel preparation: 50 µL high-dose (1 mg/g) and low-dose (0.5 mg/g)	Inhibition of the immediate early phase of infection, by determining a dysregulation of viral UL8	↓ Lesions skin	Shan et al. (2021)
*Carotenoids*						
	*Crocus sativus* L	CVB3	Male BALB/c mice, 2.5 and 5 mg/kg/day	Increasing of survival, attenuation myocardial necrotic lesions decreasing viral replication	↓ IL-17 ↓ ROCK2 expression ↓ Virus replication	Qin et al. (2021)
*Saponins*						
	*Hydrocotyle sibthorpioides* Lam	HBV	Ducks, 10 and 20 mg/kg	Inhibition of virus promoter activities	↓ Serum HbsAg ↓ Serum HbeAg ↓ Virus replication ↓ Serum ALT ↓ Serum AST	Huang et al. (2013b)
	*Astragalus membranaceus* (Fisch.) Bunge	CVB3	BALB/c mice, 60 and 120 mg/kg Sprague–Dawley rats, 100 mg/kg	Not specified	↓ Morbidity ↓ Mortality ↓ Virus titers ↓ Necrosis and mononuclear cell infiltration ↓ Heart weight/body weight ↑ mRNA IFN-γ	Zhang et al. (2006a)
	*Alternanthera philoxeroides* (Mart.) Griseb	HSV-2	BALB/c mice, 20 μL containing 0.1, 0.2 mg	Not specified	Suppression of herpetic lesions	Rattanathongkom et al. (2009)
	*Panax quinquefolius* L	IAV H1N1	BALB/c mice, 1 and 2 mg/mL	Interaction with hemagglutinin inducing loss of affinity for sialic receptor Inhibition of receptor binding by interacting close to the binding pocket of the hemagglutinin 1 subunit	↓ Viral titers Minimal weight loss No mortality	Dong et al. (2017)
		EV71	Mice (5, 10, and 20 mg/kg/day) models	Inhibition of viral replication	Inhibition EV71-induced viral protein-2	Kang et al. (2021b)
	*Panax quinquefolius* L	IAV H1N1	BALB/c mice viral inoculum pre-incubated with equal volumes of ginsenoside (2 mg/mL)	Not specified	Minimal weight loss No mortality	Dong et al. (2017)
	*Panax quinquefolius* L	IAV H1N1	BALB/c mice viral inoculum pre-incubated with equal volumes of ginsenoside (2 mg/mL)	Not specified	Minimal weight loss No mortality	Dong et al. (2017)
	*Panax quinquefolius* L	IAV H1N1	BALB/c mice Viral inoculum pre-incubated with equal volumes of ginsenoside (2 mg/mL)	Not specified	Minimal weight loss No mortality	Dong et al. (2017)
	*Glycyrrhiza uralensis* Fisch	IAV H2N2	BALB/c mice, 10 mg/kg	Stimulation of IFN-γ production by T cells	↓ Morbidity ↓ Mortality ↑ Survival rates Inhibition of virus growth in lung tissues Reductions in pulmonary consolidations	Utsunomiya et al. (1997)
	*Paris polyphylla* var. *yunnanensis* (Franch.) Hand.-Mazz	IAV	BALB/c mice, 5 and 10 mg/kg	Not specified	↓ Viral hemagglutination titer in lung tissue ↓ Lung tissue injury ↓ Thickening of alveolar wall and infiltrative inflammatory cells)	Pu et al. (2015)
	*Bupleurum* genus	IAV H1N1	C57BL/6 (B6) mice, 25 mg/kg	↓ Active caspase 3 ↓ Nucleoprotein	Minimal weight loss ↓ Viral titers in lung tissue ↓ Neutrophil and monocyte recruitment ↓ IL-6 ↓ TNF-α	Chen et al. (2015)
	*Potentilla anserina* L	HBV	Ducklings, 0.2 g/kg body weight/day	Not specified	Inhibition of virus DNA replication	Zhao et al. (2008)
	*Pulsatilla chinensis (Bunge)* Regel. roots	EV71	suckling mice, 200 mg/ kg/day	Regulation of Hippo and RLRs pathway	↑ INF-*β*	Kang et al. (2021a)
*Limonoids*						
	*Melia azedarach* L	IAV	BALB/c mice, 1 mg/kg	Virus inhibition by altering polymerase PA protein nuclear localization	Inhibition of viral hemagglutinin, nucleoprotein, polymerase PA protein and matrix protein 2 mRNA	Jin et al. (2019)
*Steroids*						
	*Cynanchum paniculatum* (Bunge) Kitag. Ex H.Hara	SINV	BALB/C mice 5, 50, and 100 mg/kg body weight	Suppression of the expression of viral subgenomic RNA(s)	↑ Protection of BALB/c mice from lethal SINV infection	Li et al. (2007)

Flavonoids						
Flavones						
	Standard compound, often found in plant kingdom	EV71	Newborn mice, 50 mg/kg	Not specified	↑ Survival rate	Dai et al. (2019)
	Scutellaria baicalensis Georgi	IAV H1N1	Mice, 240, 480, and 960 mg/kg/day Rats, 500 mg/kg/day	Not specified	↓ Virus titers (log10 EID50/ml) 3.8 ± 0.8	Xu et al. (2010)
	Scutellaria baicalensis Georgi	IAV H1N1	Mice, 100, 200, and 400 mg/kg/day	NA inhibition	↑ Protection (50 mg/Kg/day ribavirin + 400 mg/Kg/day baicalein: 100% protection)	Chen et al. (2011)
	Scutellaria baicalensis Georgi	IAV H1N1	BALB/c mice, 10–120 mg/kg/day	Modulation of viral protein NS1, by down-regulating IFN induction and up-regulating PI3K/Akt signaling	↓ Virus titre (40%) ↓ Viral NP transcription	Nayak et al. (2014)
		IAV H1N1	SPF ICR mice, 50, 100, and 200 mg/kg/day	NA inhibition	↑ Protection from death (60% at 200 mg/kg/day dose) and 30% at 100 mg/kg/day	(Ding et al. 2014)
		IAV H1N1	C57BL/6 mice, 1.0, 1.5, and 2.0 g/kg	Effect on IFN-γ production	↓ Virus titer ↑ IFN-γ level	(Chu et al. 2015)
		RSV	Female BALB/C mice, 50, 100, and 200 mg/kg	Inhibition of cell attachment and intracellular replication	↓ Virus titer ↓ Lung inflammation	(Shi et al. 2016)
		IAV H1N1 and H3N2	Female BALB/C mice, No dose	Suppression of miR146a	↓ Viral infection	Li and Wang (2019)
		RV	Neonatal mice, 0.15 and 0.30 mg/g		↓ Diarrhea ↓ Perianal inflammation	Song et al. (2021)
	Scutellaria baicalensis Georgi	IAV H1N1	Mice, 0.5 and 0.15 mg/kg Mice, 4, 40, and 400 mg/kg	NA inhibitory activity	↓ Lung virus proliferation	Nagai et al. (1992)
	Scutellaria baicalensis Georgi	IAV H3N2 and H2N3	Mice, 3.5 mg/kg	Not specified	↓ Virus titer	Nagai et al. (1995)
	Standard compound, often found in plant kingdom	HBV	Female BALB/c mice, 20 mg/kg	Inhibition of HBV transcription, by ERK-mediated down-regulation of HNF4α expression	↓ HBsAg serum level ↓ HBcAg serum level	Bai et al. (2016)
	Viola yedoensis Makino	DENV2	Sv/129 mice, 100 mg/kg	Inhibition of virus maturation, probably acting on furin-substrate complex	↓Viremia	Peng et al. (2017)
	Standard compound, often found in plant kingdom	EV7l	Newborn mice, 10 mg/kg	Not specified	↑ Survival rate	Dai et al. (2019)
	Citrus ssp.	HBV	Wide type (wt) male mice (C57BL/6), 15 mg/kg	Not specified	↓ Circulating HBsAg level	Hu et al. (2020)
	Scutellaria baicalensis Georgi	CVB3	BALB/c mice, 10 mg/kg	Not specified	No results	Kwon et al. (2016)
	Scutellaria baicalensis Georgi	IAV H1N1	ICR mice, 25, 50, and 100 mg/kg/day	NA inhibition and induction of IFN	↑ Survival rates	Jin et al. (2018)
	Iris tectorum Maxim	HBV	Transgenic mice model 5 and 10 mg/kg	Inhibition of HBV replication	No results	Xu et al. (2020)
	Citrus reticulata Blanco	RSV	BALB/c mice, 25, 50, and 100 mg/kg/day	Not specified	↓ Viral load	Xu et al. (2015)
Flavanone						
	Citrus spp.	IAV H1N1	Mice, 100 mg/kg/day	Modulation of MAP kinase signaling pathway via up-regulation of p38, JNK activation and down-regulating ERK activation	↓ Mortality ↓ Lung damage	Dong et al. (2014)
	Citrus spp.	IAV H1N1	Male Sprague–Dawley rats, 50, 200 or 500 mg/kg	Regulation of the inflammatory factors expression level	↓ Pulmonary inflammation Inhibition of pro-inflammatory cytokines (TNF-α, IFNα-, Il-6)	Ding et al. (2018)
	Cajanus cajan (L.) Millsp.	HSV-1	Mice, 20 and 50 mg/kg	Attacks the surface of viral lipid envelope	↓ Mortality rate: 16.7% (20 mg/kg) and 0% (50 mg/kg) Delayed lesion development in liver and spleen	Wu et al. (2011b)
	Citrus ssp.	EV71	One-day-old suckling BALB/c mice, 1, 3, and 10 mg/kg	Internal ribosome entry site inhibition	↓ Viral titers	Gunaseelan and Wong (2019)
Flavonols						
	Standard compound	IAV H1N1	Female C57BL/6 mice, 1 mg/kg	Inhibition of HA and viral NA	↓ Lung virus titers ↑ Survival rate	Hossain et al. (2014)
	Houttuynia cordata Thunb	HSV-1	Mice, 100 μM mixed with 1.0 × 107 PFU HSV-1/F	Blocking viral membrane fusion	↓ Viral load ↓ Skin lesions	Li et al. (2017)
	Standard compound, widely found in plant kingdom	IAV H1N1	Mice, 30 mg/kg	Not specified	↑ Surviving rate ↓ Body weight loss ↓ Viral load	Kai et al. (2014)
		IAV A/WSN/33 (H1N1)	Mice, 100 mg/kg/day	Activation of p38 and JNK and reinforced ERK signaling pathway activation	No results	Dong et al. (2014)
		IAV H9N2	BALB/c mice, 15 mg/kg	Inhibition of the TLR4/ MyD88-mediated NF-kB activation and MAPKs pathways	↑ Surviving rate ↓ Viral yield ↓ ROS and MDA production, ↓ Production of TNF-α, IL-1b and IL-6 ↑ Superoxide dismutase (SOD) activity	Zhang et al. (2017a)
		EV71	Newborn mice, 50 mg/kg	Not specified	↑ Survival rate	Dai et al. (2019)
	Standard compound, often found in plant kingdom	IAV	Mice, 1 mg/kg	Not specified	↑ Survival rates ↓ Body weight loss	Yoo et al. (2013)
		HSV-1/HSV-2	Mice, 2.5 or 5 mg/kg	Interaction with gD virus protein and cellular EGFR /PI3K /Akt pathway	↑ Survival rate ↓Body weight loss ↓ Infection symptoms	Li et al. (2020b)
	Standard compound, often found in plant kingdom	Mengo	ABD2F mice, quercetin 20 and 10 mg/kg + MuIFN-α/β 500 IU	Not specified	Not reported	Veckenstedt et al. (1987)
		IAV H1N1	ICR mice, 12.5 mg/kg	Not specified	↓ Susceptibility to influenza infection	Davis et al. (2008)
		RV1B	C57BL/6 mice, 0.2 mg/mouse	Not specified	< 1 Log TCID50/lung (4 days p.i.)	Ganesan et al. (2012)
		IAV H1N1	BALB/c mice, 240, 480, and 960 mg/kg/day	Not specified	↑ Survival rate: 80% (240 mg/kg) ↑ Survival rate and lung index (80%, 0.72 ± 0.07) at 960 mg/kg	Liu et al. (2016)
		HCV	Human patients, 250 to 5000 mg/day	Not specified	↓ HCV infection	Lu et al. (2016)
		RV1B	C57BL/6 mice with chronic obstructive pulmonary disease-like lung disease, 100 mg/kg	Not specified	↓ Viral load ↓ Lung inflammatory associated to viral infection	Farazuddin et al. (2018)
		EV71	Newborn mice, 3.13 µM	Not specified	↑ Survival rate	Dai et al. (2019)
		EV71	Mice, 10 mg/kg body weight	Not specified	↑ Survival rate ↓ BW loss	Dai et al. (2019)
	Hippophae rhamnoides L., Ginkgo biloba L	IAV H1N1	Mice, 1 mg/kg/day	Inhibition of HA and NA activity, reduced virus-induced ROS generation	↓ Lung virus titers	Dayem et al. (2015)
	Standard compound, often found in plant kingdom	IAV H1N1	BALB/c mice, 10 mg/kg/day	Inhibition of replication	↓ Viral titers	Kim et al. (2010)
		Ebola virus (Mayinga variant)	C57BL/6 mice and BALB/c mice, 50 mg/kg/day	Not specified	↑ Survival rate	Qiu et al. (2016)
	Polygonum perfoliatum L	IAV	Mice, 3 and 6 mg/kg	Not specified	↑ Inhibitory activity	Fan et al. (2011)
	Houttuynia cordata Thunb	IAV H1N1	BALB/c mice, 6.25 mg/kg/day	Not specified	↓ Viral titers	Choi et al. (2012)
	Laggera pterodonta (DC.) Sch.Bip. ex Oliv	EV71	Newborn mice, 5 mg/kg	Not specified	↑ Survival rate	Dai et al. (2019)
	Laggera pterodonta (DC.) Sch.Bip. ex Oliv	EV71	Newborn mice, 5 mg/kg	Not specified	↑ Survival rate	Dai et al. (2019)
Dihydroflavonols						
	Larix sibirica Ledeb	IAV H3N2	Mice, 0.2 mL (5, 10, and 20 µM)	Not specified	↓ Viral titers	Trofimova et al. (2015)
		CVB4	Mice, 75 and 150 mg/kg/day	Not specified	↓ Virus titers	Galochkina et al. (2016)
Isoflavones						
	Astragalus membranaceus var. mongholicus (Bunge) P.K.Hsiao	CVB3	Mice, 24 mg/kg body weight	Inhibition of virus replication in heart tissue	↑ Survival rate (77.7%) ↓ Symptoms of myocarditis	Zhu et al. (2009)
	Standard compound, widely found in Fabaceae family	EV71	Newborn mice, 2 and 10 mg/kg	Not specified	↑ Survival rate	Dai et al. (2019)
	Standard compound, widely found in Fabaceae family	IAV H1N1	Mice, 0.4 g/kg/day	Inhibition of the NA activity (cells)	↑ Survival rate	Wei et al. (2015)
	Pueraria lobata (Willd.) Ohwi	IAV H1N1	Mice, 50, 100, and 200 mg/kg	Not specified	↑ Survival rate ↓ Lung index	Wang et al. (2021)
Chalcones						
	Humulus lupulus L	HCV	Tupaia belangeri, 1 ml/100 g of XN plus 4% starch solution	Not specified	↓ Liver viral load	Yang et al. (2013a)
Catechins						
	Camellia sinensis L. Ktze	IAV H1N1	BALB/c mice, 40, 20, and 10 mg/kg/day body weight	Inhibition of replication cycle at, an early stage after virus adsorption to the host cell	↓ Viral pneumonia ↑ Survival rate	Ling et al. (2012)
		HCV	SCID/uPA mice, 100 mg/kg twice daily by gavage	Inhibition of viral replication	Monotherapy failed to protect from HCV infection	O'Shea et al. (2016)
		IAV H9N2	SPF female BALB/c mice, 10 mg/kg/day body weight	Inhibition of viral replication	↑ Survival rate (65%) ↓ Lung lesions	Xu et al. (2017)
		CVB3	Male BALB/c mice, 10 mg/kg/day body weight	Inhibition of viral replication	↓ CVB3-induced viral myocarditis	He et al. (2017)
		HBV	FRG mice, 50 mg/kg	Inhibition of replication	↓ HBV infection	Lai et al. (2018)
	Pithecellobium clypearia (Jack) Benth	IAV A/PR/8/34 (H1N1)	Male ICR mice, 3, 10, and 30 mg/kg body weight	Inhibition of CLK1	↓ Body weight loss, ↑ Protective effect on murine lung	Li et al. (2018)
Benzoic acids						
	Ginkgo biloba L	HSV-1	Mice, 10 Mm in hydroxyethyl cellulose (HEC) gel and polyethylene glycol (PEG)		↑ Survival rate ↓ Vesicle and erosion formation	Bhutta et al. (2021)
	Salvia miltiorrhiza Bunge	HBV	Duckling, 25, 50, and 100 mg/kg	Transcription, translation or post translational inhibition, inhibition HBV polymerase	↓ HBV DNA levels	Zhou et al. (2007)
Phenylpropanoids						
	Ligustrum purpurascens Y. C. Yan	IAV H1N1	C57BL/6 mice, 80 mg/kg body weight	ERK activation enhancement of IFN-γ production	↓ Lung lesions	Song et al. (2016)
		RSV	Five-week-old female BALB/c mice, 80 mg/kg body weight	Lower expression of RSV-G protein mRNA	↓ RSV replication ↓ Viral mRNA level	Chathuranga et al. (2019)
	Standard compound, Cinnamomum spp.	IAV H1N1	ICR female mice, 250 µg/mouse/day and 50 mg/mL water	Inhibition of viral replication	↓ Body weight loss ↓ Mortality rate	Hayashi et al. (2007)
		CVB3	BALB/c mice, 30 mg/kg	Expression level of NF-κB P65, iNOS, TLR4, NO synthase and TNF-α	↓ Expression level of inflammatory protein ↓ Mortality rate	Ding et al. (2010)
	Standard compound, Cinnamomum spp.	CVB3	BALB/c mice, 30 mg/kg	Expression level of NF- kB P65, iNOS, TLR4, NO synthase and TNF- α	↓ Mortality no reduction expression level of inflammatory protein	Ding et al. (2010)
		ZIKV	Mice, 75 and 150 mg/kg	No specified	↑ Survival rate ↓ Body weight loss ↓ Viral load	Chen et al. (2021)
	Standard compound, widely found in plant kingdom	IAV H1N1	BALB/c mice, 240, 480, and 960 mg/kg/day	Not specified	↑ Survival rates	Liu et al. (2016)
	Standard compound, Lamiaceae family	JEV	BALB/c mice, 25 mg/kg body weight	Expression level of viral proteins and inflammatory factors (TNF-α, IFN-γ, MCP-1, and IL-6)	↓ Mortality rate ↓ Expression level of viral proteins	Swarup et al. (2007)
		EV71	ICR mice, 20 mg/kg body weight	Inhibition of the early stage of viral infection	↑ Survival rate ↓ Body weight loss	Lin et al. (2019)
		EV71	ICR mice, 100 mg/kg/day	Targets EV-A71-PSGL1 and -heparan sulfate	↑ Survival rate ↑ Protective effect	Hsieh et al. (2020)
Stilbenoids						
	Standard compound, Vitis spp.	HSV	SKH1 mice, (cream containing resveratrol) 12.5 and 25%	Inhibition of viral replication	↓ HSV-induced lesion formation	Docherty et al. (2004)
		HSV	SKH1 mice, (cream containing resveratrol) 6.25, 12.5, and 19%	Inhibition of viral replication	↓ Vaginal virus replication ↓ Extravaginal lesion formation	Docherty et al. (2005)
		IAV H1N1	BALB/c mice, 1 mg/kg/day	Inhibition of viral replication probably related to the inhibition of PKC activity	↑ Survival rate ↓ Pulmonary viral titers	Palamara et al. (2005)
		RSV	BALB/c mice, 30 mg/kg/day	Inhibition of viral replication, reduction of IFN-γ production, inhibition of TLR3 signaling and M2R expression	↓ Viral replication ↓ Pulmonary inflammation, ↓ Bronchoalveolar inflammatory cell and lymphocyte infiltrates, ↓ Airway hyperresponsiveness response to methacholine following RSV infection ↓ BALF IFN-γ levels	Zang et al. (2011), Zang et al. (2015)
Tannins						
	Standard compound, Terminalia chebula Retz	RSV	BALB/c mice, 50 mg/kg	Regulation of IKK-NF-κB and P38-MAPK signal pathway	↓ Lung wet to dry index	Xie et al. (2012)
	Standard compound	EV71	Mice, 0.2, 1, and 5 mg/kg	Inhibition of viral absorption/penetration	↑ Survival rate ↓ Viral titers	Yang et al. (2013b)
	Standard compound, Punica granatum L	HSV-1	BALB/c mice, 0.4 mg/ mouse	Inhibition of Toll-like receptor signaling pathways	Not specified	Guo et al. (2015)
		HCV	BALB/c mice, 1 mM/day	Not specified	↓ Viral titers	Reddy et al. (2018)
	Phyllanthus urinaria L	HBV	HBeAg transgenic mice, 5 mg/kg body weight	Effects on immune tolerance	↓ HBeAg levels, ↑ HBeAb production in sera Recovery of the T/B cell response and of IgG-type antibody production, cytokine production ↑ CTL response	Kang et al. (2006)
	Geum japonicum L. and Syzygium aromaticum L. Merr. & L.M. Perry	HSV- 1	Balb/c mice, 0.3, 3, and 6 mg/kg; 0.3, 1, 3, 10, and 50 mg/kg; 0.3 to 50 mg/kg	Interaction with the HSV-1 DNA polymerase in the vicinity of PAA binding site	↓ Skin lesions	Kurokawa et al. (2001)
	Nephelium lappaceum Rin	EV71, MP10 strain	10-day-old ICR mice, 0.4 and 1.0 mg/kg	Not specified	↑ Survival rates	Yang et al. (2012c)
		DENV-2	BALB/c mice, dose not specified	Not specified	↓ Viral RNA load	Abdul Ahmad et al. (2019)
	Standard compound, Phyllanthus emblica L	CVB3	BALB/c mice, 4, 8, and 12 mg/kg/day	Not specified	↓ Cardiac CVB3 titers	Wang et al. (2009)
	Standard compound, Punica granatum L	EV71	ICR mice, 0.4, 1, and 5 mg/kg BW	Not specified	↑ Survival rate	Yang et al. (2012a)

Coumarines						
	Sarcandra glabra Thunb. and Siberian ginseng	IAV A/PR/8/34 (H1N1)	Mice, 10, 20, and 40 mg/kg	Modulation of AKT/MAPK pathways and immunomodulation activity	↑ Survival rate ↑ IAV induced lung inflammation ↑ Secondary acute lung injury ↓ Activation and aggregation of platelets ↓ PI3K, AKT, p38 sP-selectin, and PF4 ↓ TNF-α, IL-1β, IL-6, MIP-2	Jin et al. (2020)
	Calophyllum genus	HIV-1	Mice, 150 mg/kg twice a day and 200 mg/kg once a day	Not specified	↓ Virus replication in both intraperitoneal and subcutaneous compartments	Xu et al. (1999)
	Standard compound	HBV	Mice, 10 and 20 mg/kg	Inhibition of the NQO1	↓ HBx expression ↓ cccDNA ↓ HBV RNAs ↓ HBV DNA ↓ HBsAg	Cheng et al. (2021)
Lignans						
	Justicia gendarussa Burm.f	ZIKV	Mice, 1 mg/kg	Inhibition of acidification of endosomes	↓ Appearance of symptoms ↓ ZIKV-induced mortality	Martinez-Lopez et al. (2019)
		IAV H1N1 and H3N2	Mice, 10 μg in nanoparticles	Inhibition of virus entry into host cells	↑ Survival rate ↓ Viral load in mice lungs ↓ Weight loss	Hu et al. (2018)
	Schisandra chinensis (Turcz.) Baill	DENV	Mice, 5 and 10 mg/kg	Production of IFN-α protein and STAT1/2 phosphorylation	↑ Survival rate ↓ Viral titer in the brain ↓ Clinical score ↓ Body weight loss	Yu et al. (2017b)
	Arctium lappa L	JEV	BALB/c mice, 10 mg/kg	Reduction of the microglial activation and of, levels of pro-inflammatory cytokines TNF-α, IFN-γ, IL-6, and chemokine MCP-1, caspase 3 activity and the levels of ROS	↑ Survival rate ↓ Viral titer in mice brain ↓ Morbidity ↓ Viral protein expression ↓ Neuroinflammation ↓ Apoptosis	Swarup et al. (2008)
		IAV H1N1	Mice, 5 mg arctiin 1 mg + oseltamivir (0.02 or 0.05 mg	Interference with early stages after virus penetration into the host cells	↑ Sera neutralizing antibody titer ↑ Survival rate ↓ Virus production in association with oseltamivir	Hayashi et al. (2010)
	Forsythiae suspensa (Thunb.) Vahl	IAV H1N1	BALB/c mice, 10 and 20 mg/kg	Inhibition of HA expression and reduction of viral-induced inflammation, by decreasing serum levels of IL-6	↑ Survival time ↓ Lung index ↓ Virus titers ↓ Serum IL-6 ↓ HA expression ↓ Lung tissue damage	Qu et al. (2016)
Neolignans						
	Aglaia foveolata Pannell	HEV	Homozygous uPA^+^/^+^-SCID mice, 0.3 mg/kg	Not specified	↓ HEV RNA in animal faeces	Todt et al. (2018)
Diarylheptanoids						
	Alpinia officinarum Hance	IAV H1N1	Female BALB/c mice, 100 mg/kg	Not specified	↑ Survival rate ↓ Body weight loss ↓ Virus titer in mice lung	Sawamura et al. (2010)
	Alpinia officinarum Hance	RSV	Female BALB/c mice, 30 mg/kg	Not specified	↑ Pneumonia ↓ Virus titer in mice lung	Konno et al. (2013)
	Zingiberaceae family	IAV H1N1	Mice, 100 mg/kg	Link to the Wnt/β-catenin signaling and its inhibition	↑ Survival rate ↓ Body weight loss ↓ HW/BW ↓ Left ventricular weight to body weight ratio (LV/BW) ↓ TNF-α, IL-1β, and IL-6 gene expression ↓ I collagen and MMP-2 mRNA	Liu et al. (2013)
		CMV	BALB/c mice, 12.5, 25, and 50 mg/kg/day	Not specified	↓ Viral load ↓ CMV IgM level ↓ Serum AST, ALT, CK, LDH, TNF-α, and IL-6 ↓ MDA content ↑ SOD and GSH levels	Lv et al. (2014b)
		IAV	Mice, 50 and 150 mg/kg	Not specified	↑ Survival rate ↓ Lung index ↓ Pulmonary viral titer ↓ Immunity system ↑ Pulmonary histopathological changes	Dai et al. (2018b)
		IAV H1N1	Mice, 25, 50, and 100 mg/kg	Improvement of immune function and regulation of TNF-α, IL-6, and IFN-γ expression	↑ Survival rate ↑ Death protection rate	Lai et al. (2020)
Quinones						
	Hypericum perforatum L. and Hypericum triquetrifoliurn Turra	HSV-1	CD-1 mice, 50 µg/mL	Not specified	↑ Survival rate	Tang et al. (1990)
Flavolignans						
		HCV HIV	Patients, 20 mg/kg	Not specified	↓ HCV RNA levels ↓ HIV replication ↑ CD4 + cell counts during combination therapy with SIL and peginterferon/ribavirin	Payer et al. (2010)
		HCV HIV	Patients, 10, 15, and 20 mg/kg	Block of viral infection and viral production/release	↓ Viral load	Guedj et al. (2012)
		HCV HIV	Patients, 20 mg/kg	Not specified	↓ Viral load	Bárcena et al. (2013)
		HCV HIV	Patients, 20 mg/kg	Not specified	↓ HCV RNA levels	Braun et al. (2014)
		HCV HIV	Patients, 20 mg/kg	Not specified	↓ HCV RNA levels	Braun et al. (2015)
		HCV HIV	Male Sprague–Dawley (SD) rats, 5 and 10 mg/kg SB-NP	Not specified	↑ Antiviral effect respect to non-modified silibinin	Liu et al. (2017)
		HCV	Patients, 10 mg/kg	Not specified	↓ Viral load	Ferenci et al. (2008)
		HCV	Patients, 20 mg/kg	Not specified	↓ Viral load	Rutter et al. (2011)
		HCV	Patients, 1400 mg/day	Not specified	↓ Viral load	Biermer et al. (2012)
		HCV	Male patient with chronic HCV genotype 1a infection previously treated unsuccessfully with IFNa monotherapy, IFNa/RBV, and pegIFNa/RBV, 20 mg/kg	Modulation of a critical interaction with NS3/4A, and consequent inhibition of HCV replication site formation	↓ Viral load (transient)	Esser-Nobis et al. (2013)
		HCV	Patients, 20 mg/kg	Not specified	↓ Viral load	Mariño et al. (2013)
		HCV	Patients,20 mg/kg	Not specified	↓ Viral load	Knapstein et al. (2014)
		HCV	Patients, 20 mg/kg	Not specified	↓ Viral load	Rendina et al. (2014)
		HCV	Patients, 20 mg/kg	Not specified	↓ Viral load	Canini et al. (2015)
		HCV	Liver transplant patients. 20 mg/kg	Immunomodulatory activity	↓ Viral load ↑ Plasmacytoid dendritic cell (pDC)/myeloid dendritic cell (mDC) ratio	Castellaneta et al. (2016)
		HCV	Chimeric mice, 61.5, 265, and 469 mg/kg	Not specified	↓ HCV‐infected hepatocyte ↑ Anti-inflammatory and antiproliferative gene expression in human hepatocytes	Deb Roy et al. (2016)
**Silymarin**	Silybum marianum (L.) Gaernt	HCV	Patients, 125 mg	Not specified	Safe	Tanamly et al. (2004)
		HCV	Patients, 140, 280, 560, and 700 mg	Not specified	Safe	Hawke et al. (2010)
		HCV	Patients (no dose reported)	Not specified	↓ Fibrosis progression	Freedman et al. (2011)
		HCV	Patients, 420 and 1050 mg	Not specified	↓ Serum AST ↓ Serum ALT ↑ Albumin ↑ Birilubin	Fathalah et al. (2017)
		Different hepatitis viruses	Patients, 400 mg/day	Not specified	↓ Serum ALT	Jaffri et al. (2007)
	Silybum marianum (L.) Gaertn	HCV	Patients, 47 mg of silybin and 15 mg of vitamin E	Not specified	↓ Viral load ↓ Hepatic index ↓ Serum ALT and AST ↑ IL-2 ↓ IL-6	Falasca et al. (2008)
		HCV	Patients silybin 94 mg, vitamin E 30 mg, phospholipids 194 mg	Not specified	↑ PegIFN and ribavirin effect ↑ Work ability ↓ Depression and anxiety	Malaguarnera et al. (2016)
Alkaloids						
	Peganum harmala L	EV71	Mice, 12.5 μg/mL/day	Inhibition of NF-κB signaling pathway	↓ Viral replication in the gut	Chen et al. (2018)
	Rhinella genus and Fabaceae family	Rabies virus	Mice, 0.63, 1.05, and 2.1 mg/day	Inhibition of the virus entry through an apparent competition with the nicotinic acetylcholine receptor	↑ Antiviral activity	Vigerelli et al. (2018)
			Mice, 0.63, 1.05, and 2.1 mg/day	Not specified	↑ Survival rate	Vigerelli et al. (2020)
	Commelina communis L	IAV H1N1	Mice, 0.5, 1, and 2 mg/kg	Immunomodulatory effect	↑ Survival rate ↑ Viral load ↓ Acute lung injuries ↓ Inflammation	Zhang et al. (2013)
	Lycoris radiata (L’Hér.) Herb	EV71	Mice, 0.4 and 1 mg/Kg	Not specified	↑ Survival rate ↓ Number of virions in muscle tissues ↓ Muscle damage	Liu et al. (2011)
		CoV	Female BALB/c mice, 5 mg/kg	Not specified	↑ Survival rate ↓ Viral load in the central nervous system ↓ HcoV-OC43 nucleocapsid protein levels	Shen et al. (2019)
		ZIKV	Mice, 1, 5, and 10 mg/kg	Inhibition of thermally induced NS5 protein aggregation and consequent inhibition of RdRp activity	↓ Viral load in brain and liver tissue ↓ ZIKV-induced inflammatory response	Chen et al. (2020)
	Berberis genus	IAV H1N1	Mice, 0.005 g/kg	Inhibition of cytopathic effect and reduction of NA activity	↑ Survival rate ↓ Viral load in the lung ↓ Production of inflammatory compounds	Wu et al. (2011c)
		CHIKV	Mice, 10 mg/kg	Block of the three major MAPK signaling, ERK, p38 and JNK, important for the viral life cycle	↓ Viral load ↓ CHIKV-induced inflammatory disease	Varghese et al. (2016)
		IAV H1N1	Mice, 2000 mg/kg	Not specified	↓ Toxicity	(Subaiea et al. 2017)
		IAV H1N1	Mice, 20 mg/kg	Suppression of TLR7/NF‐κB signaling, virus‐induced T cell responses and expression of cytokines in lungs	↓ Viral replication in lung ↓ Body weight loss ↓ Inflammatory response	Yan et al. (2018)
		CVB3	Mice, 50 and 100 mg/kg	Survival rate and heart edema	↓ Mortality ↓ Heart edema and myocardial necrosis	Dai et al. (2021)
	Cephalis ipecacuanha	SARS-CoV-2	Patients, 3.6 mg s, 3 times per day for 10 days	Body temperature, respiratory rate, and oxygen saturation	↑ Oxygen saturation on the first day	Fan et al. (2021)
	Sophora genus	EV71	Mice, 10, 20, and 40 mg/kg	Not specified	↑ Survival rate ↓ Clinical score ↓ EV71 replication	Yang et al. (2012b)
		EV71	Mice, 7.5, 15, and 30 mg/kg	Improvement the levels of T- cells	↑ Survival rate ↑ T cells levels	Yang et al. (2015)
	Sophora genus	HBV	Mice, 100, 200, and 300 mg/kg	Not specified	↓ HBV DNAreplication ↓ Mice liver HBV antigens (HbsAg and HbcAg)	Chen et al. (2001)
			Mice, 200 mg/kg	Not specified	↑ Serum Th1 cytokines ↓ Serum Th2 cytokines	Dong et al. (2002)
			Patients, 300 mg	Not specified	↓ HBV DNA replication	Lu et al. (2003)
			Mice, 50 and 100 mg/kg	Not specified	↓ HBV DNA replication ↓ Serum HbsAg	Lu et al. (2004)
			Patients, 300 mg	Not specified	↓ Hepatic fibrosis ↓ Inflammatory activity ↓ Serum markers of hepatic fibrosis (hyaluronic acid and type III procollagenic peptide)	Mao et al. (2004)
			Patients, 0.2 g	Not specified	↓ Hsc70 mRNA ↓ HBV DNA in liver cells ↓ Drug resistance in combination with lamivudine	Wang et al. (2011)
			Patients, 600 mg once a day, then 200 mg capsule, three times a day	Downregulation of peripheral blood HBV-specific CTL surface PD-1 expression, improvement of HBV-specific CTL activity	↓ HBV DNA replication ↓ Serum HbeAg ↓ Peripheral blood HBV-specific CTL surface PD-1 expression ↑ HBV-specific CTL levels	Gu et al. (2012)
			Mice, 20 mg/kg	Not specified	↓ HBV DNA replication ↓ Serum HBV antigens ↑ CD4^+^ T-cells expression ↑ IFN-γ production	Sang et al. (2017)
		CVB3	BALB/c mice, 3.125, 6.25, 12.5, and 25 mg/kg	Regulation of cytokines	↓ Viral titer ↓ Serum NTR gene ↓ Serum TNF-γ ↓ Serum TNF-α gene ↓ LDH, CK, and CK-MB ↑ IFN-γ expression in cardiac muscle tissue of mice	Jiang et al. (2017)
		IAV H1N1	Mice, 120 mg/kg	Suppression of IAV-induced activations of TLR4, p38 MAPK, and NF-κB pathways	↑ Survival rate ↓ Body weight loss ↓ Viral titer in mice lung ↓ Lung index ↑ Pulmonary histopathological changes	Dai et al. (2018a)
	Sophora flavescens Aiton	CVB3	BALB/c mice, 20 and 40 mg/kg	↑ IFNa and IL-10 mRNA expression ↑ Resistance against virus infection, ↓ Inflammatory responses, ↓ Myocardial apoptosis and myocardial dysfunction by inhibiting TNF-mRNA expression	↑ Survival rate ↓ HW/BW ↓ Virus titers in mice heart ↓ Necrosis and mononuclear cell infiltration	Zhang et al. (2006b)
		EV71	Mice, 7.5, 15, and 30 mg/kg		↑ Survival rate ↑ T cells levels	Yang et al. (2015)
	Sophora genus	EV 71	Mice, 7.5, 15, and 30 mg/kg		↑ Survival rate ↑ T cells levels ↓ EV71 replication	Yang et al. (2015)
	Sophora genus	EV 71	Mice, 7.5, 15, and 30 mg/kg		↑ Survival rate ↑ T cells levels	Yang et al. (2015)
	Sophora genus	EV71	Mice, 7.5, 15, and 30 mg/kg	Improvement the T- cells levels	↑ Survival rate ↑ T cells levels	Yang et al. (2015)
	Castanospermum australe A.Cunn. & C.Fraser	DENV	BALB/c mice, 10, 50, and 250 mg/kg/day	Not specified	↑ Survival rate	Whitby et al. (2005)
	Ophiorrhiza nicobarica N.P.Balakr	HSV-2	Mice, 0.25 and 0.5 mg/kg/day	Not specified	↑ Survival rate ↓ Virus load in mice vaginal mucosa and brain ↓ Lesion score	Bag et al. (2013)
	Stephania cepharantha Diels	HSV-1	Mice, 10, 25, and 50 mg/kg	Not specified	↑ Survival rate ↓ Vescicles and zosteriform lesions	Nawawi et al. (2001)
		HSV-2	Mice, 0.25 and 0.5 mg/kg	↓ Recruitment of lysine-specific demethylase-1 ↓ Viral IE gene synthesis ↓ ICP4 and ICP27 expression by binding with the IE complex on ICP0 promoter	↓ Virus load ↓ Skin lesion	Bag et al. (2014)
	Isatis tinctoria L	H1N1 IV	Mice, 88 and 176 mg/kg	Improvement of mitochondria antiviral signaling, production of IFN-β and interferon inducible transmembrane 3 by reducing the expression of mitofusin 2	↑ Survival rate ↓ Virus titer in mice lungs ↓ Proinflammatory cytokines production	Luo et al. (2019)
		IAV H1N1	Eggs, 0.625, 1.25, 2.5, and 5 mg/mL		↓ Virus titer	Nie et al. (2020)
	Veratrum genus	RSV	Mice, 100 mg/kg	Reduction in viral antitermination factor M2-1	↓ Pulmonary virus titer	Bailly et al. (2016)
	Stephania tetrandra S. Moore	HSK	BALB/c mice, 15 mg/kg or 30 mg/kg	Not specified	↓Delayed-type hypersensitivity responses to HSV	Hu et al. (1997), Hu et al. (1999)
	Pancratium maritimum L	JEV	Mice, 4 and 6 mg/kg	Not specified	↑ Survival rate	Gabrielsen et al. (1992)
	Isatis tinctoria L	JEV	BALB/c mice, 1 mg/kg	Not specified	↑ Survival rate	Chang et al. (2012)
		IAV H1N1	Mice, 2.5 and 5 mg/kg	Improvement of mitochondrial antiviral-signaling protein expression and promotion of the phosphorylation of interferon regulatory factor 3 in pulmonary tissues	↑ Survival rate ↓ Morbidity ↓ Body weight loss ↓ Pulmonary edema ↓ Lung index ↓IL-1β, IL-6, and TNF-α production in mice lung ↑ IL-10 production in mice lung	Jie et al. (2017)
	Stefania and Corydalis genus	JEV (GP-78)	Mice, 2 mg/kg	Not specified ↑ Survival rate ↓ Viral load in mice brain	↓ Viral protein expression ↓ TNF-α, IFN-γ, MCP-1, and IL-6	Lixia et al. (2018)
	Aconitum genus	HSV-1	T6S‐mice, 1 μg/kg	Not specified	↑ Survival rate	Kobayashi et al. (1998)

### Terpenes

Terpenes are one of the largest classes of specialized metabolites, comprising over two-thirds of compounds. They are biosynthesized by fungi, plants, and bacteria and are the major essential oils components. Terpenes consist of isoprene unity based on which it is possible to distinguish monoterpenes, sesquiterpenes, diterpenes, sesterpenes, triterpenes, etc. (Perveen 2021). Terpenes have attracted particular attention for their antiviral action, specifically against the influenza virus but also against hepatitis viruses and herpes simplex viruses. The antiviral may be related whit their structure; e.g. it was demonstrated that terpenoid skeleton spatial arrangement, when linked with an α-methylene-γ-moiety, determines an increase in antiviral activity (Hwang et al. 2006). This section will discuss what is known about the pre-clinical antiviral activity of terpenes to date.

#### Monoterpenes and Iridoids

Monoterpenes consist of two isoprene units (C_10_), and their structure is extensively related to the antiviral and anti-inflammatory activity (Perveen 2021). Specifically, **1,8-cineole**, or eucalyptol, a bicyclic monoterpene from *Eucalyptus* spp. (Myrtaceae) essential oil has shown interesting activity against IAV, both alone and in combination with Oseltamivir, a known antiviral drug that acts as a neuraminidase enzyme inhibitor blocking the virus diffusion. Several studies have demonstrated that influenza virus infections are markedly related to a systemic inflammation characterized by the so-called “cytokine storm,” which is supposed to increase mortality (Chan et al. 2005). NF-*κ*B is involved in regulating chemokine and cytokine release during influenza; thus, the inhibition of this protein complex may block virus replication and thus, inflammatory response. In vitro investigation demonstrated 1,8-cineole anti-inflammatory activity and inhibitory effect of the nuclear factor NF-*κ*B (Juergens et al. 1998; Lima et al. 2013), which was corroborated by in vivo investigation on infected mice. In fact, the administration by oral gavage of 1,8-cineole (30, 60, and 120 mg/kg) resulted in a down-regulation of IL-1*β*, IL-6, TNF-*α*, and IFN-*γ* levels in the lung and IL-4, IL-5, IL-10, and MCP-1 in nasal lavage fluids, similar to oseltamivir (10 mg/kg). In addition, a modest reduction in lung ICAM-1, VCAM-1, and NF-*κ*B p65 expression was also demonstrated. Thus 1,8-cineole can protect mice from IAV challenges, suppressing virus production and inflammatory responses (Li et al. 2016). These results were confirmed by another in vivo investigation on female BALB/c mice infected by IAV/Victoria/3/75 (H3N2) strain, where 1,8-cineole (30, 60, and 120 mg/kg/day) was orally administrated in association with oseltamivir (0.1, 0.2, and 0.4 mg/kg/day). In this case, the combination resulted in a higher protective effect than monotherapy with either 1,8-cineol or oseltamivir (Lai et al. 2017). The anti-inflammatory effect also underlined the anti-IAV action of **paeoniflorin,** a monoterpene glucoside from *Paeonia lactiflora* Pall. (Paeoniaceae), on influenza virus A/FM/1/47 intranasally infected mice. Paeoniflorin (50 and 100 mg/kg/day, orally administrated) might indeed reduce the production of pro-inflammatory cytokines and lung collagen deposition by down-regulating the expression levels of NF-*κ*B p65, αv*β*3, TGF-*β*1, p38MAPK, and p-Smad2 in lung tissue. Paeoniflorin also has an anti-inflammatory and antifibrotic effect, reducing acute lung injury related to IAV infection (Yu et al. 2021). Another monoterpene phenol, **carvacrol,** from *Mosla chinesis* Maximim (Lamiaceae), was demonstrated to protect from the IAV-induced excessive inflammation in C57BL/6 infected mice (50 mg/kg/day) by regulating the innate immune response (Zheng et al. 2021). Similarly, iridoids, small terpene derivatives falling into the group of monoterpenes, showed anti-inflammatory and antiviral activity. It was seen that **geniposide**, an iridoid glycoside from *Gardenia jasminoides* J. Ellis (Rubiaceae), successfully blocked cellular injury induced by the pandemic influenza A/Jiangsu/1/2009 (H1N1) virus and attenuated virus-induced severe lung damage, alleviated viral titers, and decreased mortality (Guo et al. 2020; Zhang et al. 2017b). Geniposide intraperitoneal administration to pandemic A/Jiangsu/1/2009 (H1N1) influenza virus-infected mice (5, 10, and 20 mg/kg/day) was reported to inhibit virus-induced alveolar haemorrhage and neutrophil infiltration in lung tissues and to decrease inflammatory mediators such as TNF-*α*, INF-*γ*, IL-4, IL-6, and IL-10 (Zhang et al. 2017b). As in vitro demonstrated, geniposide (320, 160, 80, and 40 μg/mL) anti-IAV effect should be PACT-dependent. This molecule may indeed interfere with the interaction between the double-stranded RNA-binding protein PACT and IAV polymerase, leading to IAV-host infection prevention (Guo et al. 2020).

Apart from IAV infection, several iridoid derivatives also showed remarkable activity against encephalomyocarditis (EMCV, Picornaviridae), Semliki forest virus (SFV, Togaviridae), and hepatitis B virus (HBV). Iridoid glycosides like **arbotristoside A** and **C**, isolated from *Nyctanthes arbor-tristis* L. (Oleaceae) and named arbotristosides, showed interesting antiviral activity during in vivo investigation on CDRI SWISS mice infected with EMCV and SFV*.* Arbotristoside A and C (0.5 mg/mouse) showed promising results in SFV- and EMCV-infected mice with a protection rate of 60 and 50% and an average survival time of 6.8 and 6 days. Similarly, crude ethanolic extract showed significant antiviral activity at 10 and 20 mg/mouse, while the higher dose (40 mg/mouse) was toxic (Rathore et al. 1990). Likewise, a more recent in vitro investigation demonstrated a pronounced inhibitory activity against SFV and EMCV for either *Nyctanthes arbor-tristis* ethanolic extract, *n*-butyl fraction, and arbotristoside A and C. The *n*-butyl fraction and ethanolic extract, at a daily dose of 125 mg/kg body weight, also preserved EMCV-infected mice by 60% and 40%, respectively, from SFV infection (Gupta et al. 2005). On the other hand, **oleuropein**, a secoiridoid glycoside from *Jasminum officinale* L. var. *grandiflorum* (Oleaceae), demonstrated anti-hepatitis B activity in both in vitro and in vivo investigations. Specifically, oleuropein blocked the HBV antigens secretion (HBsAg) dose-dependently in infected HepG2 2.2.15 cells (IC_50_ = 23.2 μg/mL). It was hypothesized that oleuropein might directly alter HBsAg gene transcriptional machinery in the cell as glucocorticoid does, or it may act by targeting the cell membrane leading to the delivery to the nucleus of an inhibitory signal as insulin does. This antiviral activity was corroborated in vivo, as the intraperitoneal administration of oleuropein (80 mg/kg twice daily) to DHBV-infected ducks reduced viremia. However, the real mechanism by which oleuropein could determine this anti-HBV effect remains unknown (Zhao et al. 2009a).

#### Sesquiterpenes

Sesquiterpenes consist of three isoprene units (C_15_) and are known to have several biological activities like antiviral, antifungal, antibacterial, antitumoral, anti-inflammatory, and insecticidal (Perveen 2021). As previously mentioned, the antiviral activity of terpenes is significantly improved by the fusion with the α-methylene-γ lactone moiety (Hwang et al. 2006), as demonstrated by the number of studies attesting to the high activity of sesquiterpene lactone against IAV. In particular, **atractylon**, a sesquiterpene lactone from dried roots of *Atractylodes macrocephala* Koidz. (Asteraceae), exhibited interesting antiviral activity against IAV A/PR/8/34 virus (H1N1) and A/Shenzhen/203/2001 (H3N2) in vitro (IC_50_ = 8.9 and 9.4 μg/mL, respectively)*,* showing greater antiviral action than ribavirin (IC_50_ = 14.2 μg/mL), a known antiviral drug*.* It seems that atractylon inhibited agglutination formation by IAV, suggesting that this sesquiterpene may act by blocking virus absorption by host cells or inhibiting virus replication. These in vitro data were further elucidated in vivo on male ICR mice (10 and 40 mg/kg/day, intragastrically administrated) since an increase in survival rate and reduction of lung index and virus load was observed. Furthermore, it was demonstrated that the administration of atractylon increased serum levels of IFN-β, which plays an important role in influenza infection but decreased the levels of other pro-inflammatory cytokines such as IL-6, TNF-α, and IL-1β. Moreover, upregulation of expression of TLR7, MyD88, TRAF6, and IFN-α mRNA and downregulation of NF-κB p65 protein expression in the lung tissues was observed. Hence it is possible to affirm that the atractylon effect on IAV infection should be partially due to the activation of the TLR7 pathway to increase IFN-β expression and NF-κB p65 inhibition (Cheng et al. 2016). A greater anti-viral effect than ribavirin was also demonstrated for pseudoguainolides containing an *α,β*-unsaturated cyclopentenone moiety from *Centipeda minima* (L.) A. Braun & Asch. (Asteraceae). Specifically, **brevilin A** exhibited marked in vitro anti-IAV/Puerto Rico/8/34 H1N1 activity on MDKC cells since it prevented the viral life cycle late state and blocked viral replication by inhibiting M2 protein synthesis; at the concentration of 8 μM, the brevilin A and ribavirin inhibition rates were 100% and 38%, respectively (Zhang et al. 2018). This is a promising result since, to date, M2 ion channel inhibitors are among the two classes of antivirals approved by the FDA; examples of synthetic M2 inhibitors are rimantadine and amantadine. The brevilin A proposed mechanism of action was further confirmed and integrated by subsequent in vitro and in vivo investigations. In vitro studies demonstrated that this sesquiterpene lactone might inhibit viral replication through three different mechanisms: the prevention of vRNA synthesis, viral mRNA expression, viral ribonucleoproteins nuclear exportation, and matrix and nonstructural protein expression (Zhang et al. 2019). Matrix proteins comprise the M2 protein, a proton-selective channel protein exposed on the virion surface, and M1, a structural protein matrix located underneath the viral envelope. Similarly, for the nonstructural protein NS, it is possible to distinguish the NS1 protein and the NS2 protein. NS1 with the nuclear export protein and M1 is responsible for vRNPs nuclear exportation during the virus's life cycle. On the other hand, NS2 plays a key role in modulating immune responses of the host through interferon (IFN)-antagonist actions to enhance efficient viral replication (Lin et al. 2020). Hence by inhibiting these proteins' expression, brevilin A prevented virus replication. All these mechanisms were in vivo corroborated since brevilin A intraperitoneal administration (25 mg/kg/day) to A/PR/8/34 H1N1-infected female BALB/c mice resulted in a retard time to death with 50% of surviving after 14 days post-infection (Zhang et al. 2019).

Sesquiterpene lactones from *Curcuma longa* L. (Zingiberaceae) essential oil like **germacrone** and **curdione** showed anti-viral activity against IAV (Li et al. 2020a). Specifically, germacrone, like the other molecules discussed, exerted its antiviral action during the IAV (A/PuertoRico/8/34) first step of infection. In fact, as demonstrated in vitro*,* in addition to its ability to prevent viral entry/attachment to the host cell, it inhibited the expression of viral protein and viral RNA replication. Contrarily, antiviral activity was not exerted if germacrone was inoculated after 4 h of infection, indicating that late stages (i.e., assembly and release) were not affected (Liao et al. 2013). Underlying the prevention of viral replication could be the ability of germacrone to decrease the expression of TAP1, a multidrug resistance protein/TAP subfamily member which could be induced by IAV, leading to its increased replication (Li et al. 2020a). This mechanism of action was traduced on in vivo model of infected mice (50 or 100 mg/kg) with a lung index score reduction and an alleviation of lung tissue pathological injury resulting in a considerable delay in mortality. A combination study in mice was conducted using 100 mg/kg germacrone and 1 mg/kg oseltamivir to evaluate a possible synergistic action demonstrating that the combination treatment produced 90% survival, whereas 50% and 40% survival rates were obtained when germacrone and oseltamivir were used alone, respectively (Li et al. 2020a; Liao et al. 2013). If these molecules acted by inhibiting only the first steps of viral infection, a tricyclic sesquiterpene from *Pogostemon cablin* (Blanco) Benth. (Lamiaceae) essential oil*,* the **patchouli alcohol**, seemed to be active when added before infection and during adsorption determining a virus multiplication reduction. The earliest in vitro studies on this molecule indicated that it inhibited the NA protein known to play a key role in releasing new virions from infected host cells (Wu et al. 2011a). However, a more recent investigation showed no significant inhibition of H1N1 viruses’ NA activity. Similarly, the patchouli alcohol did not significantly block agglutination by inhibiting virus HA as the sesquiterpene atractylon did. Patchouli alcohol seemed to act by directly inactivating IAV and inhibiting crucial early stages after virus absorption since intracellular PI3K/Akt and ERK/MAPK signaling pathways could be involved in patchouli alcohol anti-IAV action (Yu et al. 2019). This is an interesting result since the intracellular PI3K/Akt signaling pathway was implied in augmented virus replication and could be linked with either RNA or DNA viruses lytic infection, including IAV (Kindrachuk et al. 2015). Inhibitors of PI3K or its Akt downstream signal were demonstrated to block virus entry and replication. Patchouli alcohol was found to inhibit both PI3K and Akt protein in cells infected by IAV, indicating that it inhibited PI3K/Akt signaling pathway activation leading to the inhibition of virus infection and replication (Yu et al. 2019). In addition, in vivo investigation on mice infected with the lethal dosage of H2N1 showed that patchouli alcohol intragastric administration (20, 40, and 80 mg/kg/day) exerted viral protection not only through an anti-inflammatory activity but also by enhancing immune responses, as confirmed by the increase in CD3+ and CD4+ T cell percentages and CD4+ /CD8+ ratio, and decreased CD8+ T cell levels (Li et al. 2012). Finally, it was seen that the molecule was more active than oseltamivir as its intranasal low dose administration (20 µg/day) was found to have a comparable antiviral effect to the synthetic drug orally administered (10 mg/kg/day) (Yu et al. 2019). As previously mentioned, another protein highly implicated in the initiation of virus infection is HA, a trimeric surface glycoprotein of IAV. Each HA monomer comprises 2 subunits, HA1 containing the receptor-binding domain and HA2 involved in the fusion between the virus envelope and the cellular membrane (Shental-Bechor et al. 2002). It was seen that the sesquiterpene derivative, **stachyflin**, from *Stachybotrys* spp*.* RF-7260 (Stachybotryaceae), exerted good in vitro antiviral activity against IAV (H1N1 and H2N2) with an IC_50_ of 0.003 µM in MDBK cells (Taishi et al. 1998). This activity seems closely related to the inhibition of HA, resulting in the impossibility of assuming the enzyme conformational change necessary for the virus fusion with the cell membrane (Yoshimoto et al. 1999). Specifically, it was demonstrated that stachyflin inhibited H2, H2, H5, and H6 influenza viruses’ growth by binding HA2 active site, and so preventing the activation of HA and the initial steps of viral infection (Motohashi et al. 2013). However, despite this promising in vitro antiviral activity, when orally administered to mice (20 mg/mouse), stachyflin demonstrated less activity due to its low gastrointestinal bioavailability. Contrarily, intraperitoneally treatment (2 mg/mouse) showed a percentage of virus inhibition in the lung of 64% (Yagi et al. 1999). Hence, different vehicles were studied to improve stachyflin oral absorption and polyethylene glycol 400 (PEG400) demonstrating to improve oral bioavailability and in vivo anti-influenza effect (Motohashi et al. 2013; Yagi et al. 1999). In fact, when stachyflin was administred to mice with a PEG400 solution it was seen a maximum plasma concentration of 1.68 ± 0.90 in healthy mice and % virus inhibition in lung of 60 ± 7% in infected ones (Yagi et al. 1999).

#### Diterpenes

Diterpenes, terpenes consisting of four isoprene units (C_20_), form a large class with more than 10.000 different structures. (Perveen 2021). Most diterpenes are specialized metabolites and may have roles in the ecological interactions of plants and contribute to plant fitness. These compounds are found in plants, algae, fungi, animals, and coral, and an increasing number of these terpenes have shown interesting activities both in vitro and in vivo*,* including antiviral. Labdane diterpenes’ skeletal structure can be fragmented into a fused decalin system (C1–10) and a branched six-carbon side chain (C11–16, with C16 attached to C13) at C9. These types of diterpenes comprise Andrographolide and 14-Deoxy-11,12-didehydroandrographolide, which were reported to have anti-influenza, anti-HIV, and anti-DENV activity. **Andrographolide,** a bitter diterpenoid typical of *Andrographis paniculata* (Burm.f.) Nees (*Acanthaceae*), was found to inhibit different influenzas virus strains like H5N1, H9N2, and H1N1 (Chen et al. 2009). The proposed mechanism of action involved the inhibition of the RLRs signaling pathway induced by the virus (Yu et al. 2014). RLRs were able to recognize the influenza virus RNA structure leading to the signal transduction pathway activation, including NF-*κ*B, and the up-regulation of chemokines, anti-apoptotic proteins, and growth factors gene expression (Moore and Ting 2008). Hence, andrographolide, by inhibiting this pathway, may ameliorate IAV infection. Moreover, this diterpene was also demonstrated to act downstream RLRs by down-regulating crucial inflammatory factors like the NF-*κ*B signal pathway and JAK/STAT signals (Ding et al. 2017). These data corroborated results obtained by another investigation demonstrating that andrographolide inhibited NF-*κ*B activation dose-dependently by linking the p50 reduced cysteine 62, thereby preventing the binding of NF-*κ*B to DNA (Hidalgo et al. 2005). Despite these promising results, andrographolide was less effective than approved drugs such as oseltamivir or ribavirin in the immediate treatment. However, it is also true that licensed anti-viral drugs need to be administrated immediately after infection since a delay in the administration (4 days post-infection) led to oseltamivir or ribavirin activity like a placebo. In contrast, andrographolide was effective when used as immediate and delayed treatment due to its peculiar and multiple mechanisms of action as demonstrated by in vivo investigation, where an increase in survival rate and a decrease in the lung pathology, virus load, and inflammatory cytokine expression was observed when administered orally (100 to 200 mg/kg/day twice daily for 7 days) and intraperitoneally (10 mg/kg/day) (Chen et al. 2009; Ding et al. 2017). Similarly, an analogous and metabolite of andrographolide, the **14-deoxy-11,12-didehydroandrographolide**, from *Andrographis paniculata* (Burm.f.) Nees (Acanthaceae), exhibited a good antiviral activity again H1N1, H3N2, and H5N1. As for andrographolide, its analogous decreased the upregulated proinflammatory cytokines/chemokines expression induced by IAV infection. However, this molecule differed from andrographolide in inhibiting viral replication by limiting the exportation from the nucleus to the cytosol of vRNP complexes required for the final IAV progeny virions assembly and release (Cai et al. 2015). The anti-inflammatory and anti-viral activity of 14-deoxy-11,12-didehydroandrographolide was in vivo corroborated since it alleviated lung histopathology, strongly inhibited the expression of pro-inflammatory chemokines and cytokines, and reduced lung virus titres in mice lethally challenged with IAV at the doses of 1000 mg/kg/day (intragastrically administrated) (Cai et al. 2016). As previously stated, labdane diterpenes also demonstrated antiviral activity against HIV and DENV. Specifically, in vitro investigations have shown that either andrographolide or its analogue reduced the p24 antigen amount on MT2 cells proposing themselves as potential anti-HIV molecules (Niranjan Reddy et al. 2005). The p24 antigen is indeed the main viral marker for HIV infection detection since it appears 2 weeks after HIV infection as a consequence of an initial viral replication burst that is associated with high viremia levels during which the patient is acutely infectious (Bystryak and Acharya 2016). Further, andrographolide was reported to inhibit cell-to-cell transmission, viral replication, and syncytia formation in HIV-infected cells (Chang and Yeung 1988; Yao et al. 1992). The proposed mechanism of action is related to a decrease in HIV-infected cells’ c-Mos expression, a protein required for HIV replication, and inhibition of MAPK and proteins involved in apoptotic regulation (p53, c-myc, Bxl-2, Bax, Bcl*xl*) expression. The inhibition of these apoptotic regulatory proteins’ expression is indeed involved in inhibiting the formation of HIV-envelope protein-mediated syncytia (Ma et al. 1997). Hence andrographolide might act by multiple antiviral actions, thereby inhibiting the dysregulated signal transduction pathways essential for viral replication and the T cell cytopathicity induced by HIV. The potential application of this diterpene as anti-HIV was also demonstrated in a phase I clinical trial, where andrographolide (10 mg/kg body weight three times per day) determined a significant increase in CD4+ lymphocyte counts in HIV-positive patients, especially at week 6 of treatment. This effect was comparable to that seen in other clinical trials involving synthetic drugs like zidovudine, didanosine, lamivudine, or ritonavir used in combination or in monotherapy (Calabrese et al. 2000). Finally, andrographolide showed antiviral activity against dengue virus (DENV) replication in vitro, and this activity may be related to an increase of HO-1, an antioxidant enzyme strongly related to an inhibition of DENV replication. This assumption was confirmed by the fact that by using the HO-1 inhibitor SnPP, the andrographolide anti-DENV activity was attenuated. Further, in vivo investigations also corroborated the anti-DENV activity of this diterpene since its intraperitoneally administration to infected mice (10 mg/kg) increased the survival rate by 60% and decreased viral titer and illness (Tseng et al. 2016).

Apart from labdane diterpenes, tetracyclic diterpenoids, such as scopadulcic acid B and aphidicolin, also had an antiviral activity principally against HSV-1 and HSV-2. Specifically, **scopadulcic acid B**, from *Scoparia dulcis* L. (Scrophulariaceae), showed prominent activity against HSV-1 (IC_50_ 0.012 ± 0.002 µg/mL). In vitro experiments have suggested that scopadulcic acid B interferes in the early stages of virus replication, such as the fusion of the viral envelope with plasmatic membrane, transport of the capsids through the nuclear pores, and release of the DNA into the nucleus, and that it has no virucidal effect. Based on these results, scopaldulcic acid B was also tested intraperitoneally (20 and 200 mg/kg) and orally (20 and 100 mg/kg) on female golden hamsters to evaluate the HSV-1 corneal infection. A reduction of facial lesions and increased life expectancy in animals was demonstrated, confirming the potential use of this molecule as an anti-HSV-1 agent (Hayashi et al. 1988). Similarly, **aphidicolin,** a diterpene-type antibiotic produced by *Cephalosporium aphidicola* Petch, inhibited the growth of either HSV-1 or HSV-2, in vitro and in vivo (Bucknall et al. 1973). In vitro results demonstrated that aphidicolin, administered on infected human embryonic lung cells, inhibited the growth of HSV-1 and HSV-2 with an IC_50_ value of 0.2 µg/mL. Furthermore, aphidicolin was tested in vivo to evaluate its efficacy on ocular herpetic keratitis and herpetic encephalitis. Aphidicolin was applied at different concentrations (1 and 10 mg/mL) on the eyes of HSV-1-infected rabbits. The highest concentration showed a reduced virus challenge and maximal lesions score comparable to 5-iodo-2-deoxyuridine (1 mg/mL). Moreover, aphidicolin at 10 mg/mL could also reduce the severity of keratitis in the eyes of rabbits infected with a 5-iodo-2-deoxyuridine-resistant strain (Bucknall et al. 1973). It is known that HSV encodes for a conserved DNA polymerase which is necessary for viral genome replication. This DNA polymerase is an essential therapeutic target; in fact, the licensed HSV therapies link the HSV polymerase when it is in the DNA-bound state, thereby blocking virus replication. Aphidicolin acted as a nucleotide competing inhibitor, bounding the polymerase active site and blocking it in the open conformation resulting in the inability of the virus to replicate its DNA (Baranovskiy et al. 2014; Hayes et al. 2021).

#### Sesterterpenes

Sesterpenes, the class of terpenoids consisting of five isoprene units (C25), are usually found in fungi, insects, plants, and marine organisms and have several biological activities like nematocidal, cytotoxic, anti-inflammatory, anti-viral, and antimicrobial action. In particular, a fungal sesterterpene from *Bipolaris oryzae* (Breda de Haan) Shoemaker (Pleosporaceae), **3-anhydro-6-hydroxy-ophiobolin A**, showed promising antiviral activity against IAV, especially regarding A/WSN/33 strain. Preliminary in vitro experiments demonstrated that L435-3 (0.5 μmol/L) reduced viral titers, hemagglutinin, and nucleoprotein protein levels, suggesting an evident inhibition of IAV replication. These results were also confirmed by in vivo experiments since its intranasal administration to WSN-infected Balb/c mice (0.3 mg/kg/day) showed a reduction of symptoms severity, including pulmonary lesions, inflammation, and atrophy of the thymus and spleen. In addition, the treatment with L435-3 significantly reduces the viral titers in the lungs of WSN-infected mice and the protein levels of hemagglutinin and nucleoprotein, corroborating in vitro investigations. Investigations to understand the L435-3 mechanism of action revealed an increase in IL-28, ISG15, and ISG20 expression levels in either IAV-infected cell lines or mice. Hence, L435-3 increased the expression level of type III interferons and various interferon-stimulated genes (ISGs), thereby inhibiting IAV replication (Wang et al. 2016).

#### Triterpenes

Triterpenes, one of the major classes of specialized metabolites, are formed by six isoprene units (C_30_) and have demonstrated promising activity against hepatitis virus infections. Hepatitis virus is known to be the main cause of inflammatory liver disease; it is possible to distinguish five different types of hepatitis viruses represented by hepatitis A (HAV), B (HBV), C (HCV), D (HDV), and E (HEV) all of which derived from different virus families. Different triterpenes showed anti-hepatitis B, C, and E activity. **Methyl helicterate**, a triterpenoid isolated from *Helicteres angustifolia* L. (Malvaceae), demonstrated anti-HBV activity in vitro and vivo. The compound markedly decreased HBsAg and HBeAg secretion, HBV DNA and cccDNA levels, and viral RNA. The first intermediate generated when HBV enters the hepatocytes is cccDNA, whose presence signals intracellular HBV replication and infection initiation. The permanency of cccDNA in hepatocyte nuclei after anti-viral drug suspension is thought to be the main contributor to hepatitis B recurrence. For this reason, nuclear cccDNA levels are an important index to evaluate as a predictor of the new anti-HBV agent’s effective antiviral activity. Methyl helicterate reducing effect on liver cccDNA and total viral DNA levels was also in vivo confirmed on DHBV-infected ducks (50 or 100 mg/kg of compound orally administered). Moreover, in animal models, it was also possible to observe histopathological improvement, demonstrating that methyl helicterate exhibited protective effects on liver injury induced by HBV. These effects are more important than that seen for Lamivudine, a licensed drug used for HBV infection treatment, administered at the same doses (100 mg/kg) (Huang et al. 2013a). **Betulinic acid**, a pentacyclic lupane-type triterpene isolated from *Anemone chinensis* Bunge (Ranunculaceae), acts against HBV infection with a different mechanism of action. Specifically, it seemed to inhibit a host antioxidant enzyme, the manganese superoxide dismutase, involved in scavenging superoxide anions to generate hydrogen peroxide. For biological systems, SOD2 overexpression is favourable for protecting against cell damage mediated by ROS, but it may improve the possibility of a viral infection by stimulating virus replication. Therefore, inhibiting SOD2 activity could be a target to reduce HBV invasivity. Betulinic acid has been demonstrated to inhibit SOD2 expression levels resulting in reduced HBsAg, HBeAg, HBV DNA levels, and HBV X protein (HBx) expression (Yao et al. 2009). The last result is important since HBx is highly involved in HBV-induced hepatocellular carcinoma by promoting cell cycle progression, inactivating negative growth factors, and downregulating the tumor suppressor gene p53(Kew 2011). Betulinic acid anti-HBV mechanism of action involving SOD2 is confirmed by the evidence that an induced SOD2 overexpression during compound treatment totally abrogated its antiviral activity while a SOD2 knockdown mimicked the compound anti-HBV effect. Corroborating this data are those from in vivo investigations by which it was possible to observe that betulinic acid (2 mg/kg/day) increased the superoxide anion levels in the liver, the main organ affected by HBV infection. In fact, little effect was seen in aorta tissues, and no effect was observed in kidney and brain tissues, demonstrating that betulinic acid could be a good anti-HBC drug by specifically acting on hepatocytes (Yao et al. 2009). Betulinic acid was also investigated for its anti-IAV activity, and it was seen that it did not inhibit IAV replication as done for HBV but reduced lung damage. In fact, its intraperitoneal administration (10 mg/kg/day) to infected mice showed anti-inflammatory properties by reducing INF-γ levels, leading to improved viral-related inflammatory lung diseases (Hong et al. 2015). While the compounds discussed above act mainly by inhibiting viral replication, the **schizandronic acid**, a tetracyclic triterpenoid from *Schisandra sphenanthera* Rehd. et Wils (Schisandraceae), acts by inhibiting HV entry into the host cell. Specifically, schizandronic acid antiviral activity was studied against HCV. It is known that the entry of this virus throughout hepatocytes involves several factors comprising either the host cell machinery or viral envelope glycoproteins (Barth et al. 2003; Syed et al. 2014). The low-density lipoprotein receptor and glycosaminoglycans showed to play an important role in concentrating HCV particles on hepatocytes surfaces. The compound structure looks like cholesterol, an essential element for HCV cell entry; hence, it was thought that this molecule acted by altering the host cell membrane’s fluidity. This mechanism of action might be responsible for the antiviral activity in mice treated with schizandronic acid (5 mg/kg/day via intraperitoneal injection) 2 weeks before virus inoculation and one week later. Compared to the control, HCV infection and viral RNA levels decreased in treated mice. Based on this data, schizandronic acid could be considered a lead compound for developing entry inhibitors to be combined with direct-acting antivirals currently used in treating HCV infection (Qian et al. 2016).

**20(*S*)-protopanaxtriol**, one of the most important triterpenes extracted from the roots of *Panax notoginseng* (Burk.) F.H. Chen (Araliaceae) was studied for its antiviral activity against coxsackievirus B3 (CVB3), a virus linked to viral myocarditis. This triterpene seems to exert its antiviral properties through anti-inflammatory and anti-apoptotic activity. The oral administration of 20(*S*)-protopanaxtriol to mice (100, 200 and 400 mg/kg/day) significantly reduced heart viral titer, lowering myocardium damage and mononuclear cell infiltration (Wang et al. 2012). Similarly, **celastrol**, a quinone methide triterpene from the root of *Tripterygium wilfordii* Hook. f. (Celastraceae), showed an indirect antiviral activity against DENV infection. It was indeed observed that this molecule inhibits DENV replication by upregulating IFN expression and activating the downstream Jak-STAT signaling pathway leading to an increase in IFN-α-2, IFN-α-5, OAS1, OAS2, and OAS3 gene expression levels (Yu et al. 2017a). Finally, **lupeol,** a common pentacyclic triterpene from the roots of *Carissa spinarum* L. (Apocynaceae), showed promising antiviral activity against the HSV-1 strain resistant to acyclovir using in vivo models (5, 10, and 20 mg/kg/day, orally administrated). Moreover, its mechanism of action needs to be investigated (Tolo et al. 2010). The anti-HSV-1 mechanism is instead clear for **oleanolic acid** (EC_50_ = 6.8 mg/mL), a pentacyclic triterpenoid found in different natural products, which had been demonstrated to prevent the immediate-early phase of infection. An in vivo investigation showed that oleanolic acid acted by inhibiting viral UL8, an essential component of the viral helicase involved in HSV-1 replication; as a result, an amelioration of skin lesions was observed in infected mice treated with 50 μL of a gel preparation containing 1 mg/g and 0.5 mg/g oleanolic acid (Shan et al. 2021).

#### Saponins

Saponins, chemically designated as steroid and triterpenes glycosides, consist of non-polar aglycones bonded to a variable number of monosaccharides. The term saponin derives from the Latin “Sapo” and refers to its soap-like behavior in water attributable to the combination of polar and non-polar structural moieties. These active molecules are found in several organisms but are most commonly found in plants, producing them as a defence mechanism (Sharma et al. 2021). Regarding saponin antiviral action, it seems to act against different viral strains. Oleanane-type triterpenoid saponins like Saikosaponin A, Glycyrrhizin, and Polyphylla saponin I have shown an interesting anti-IAV activity induced by a reduction in inflammation. Specifically, **saikosaponin A**, from *Bupleurum* genus (Apiaceae), attenuated in vitro the IAV replication (IC_50_ of 1.98, 2.21, and 2.07 μM for H1N1 (PR8), H9N2, and H5N1, respectively) by inhibiting the NF-*κ*B signaling pathway and caspase 3 associated with the cytosol release of vRNP. As mentioned before, NF-*κ*B activation is a fundamental requisite for IAV (H1N1 (PR8), H9N2, and H5N1) replication but is also responsible for the high inflammatory process associated with IAV infection. Saikosaponin A subcutaneous administration (25 mg/kg/day) to infected mice determines an attenuation of lung monocyte and neutrophil recruitment and a decrease in the lung pro-inflammatory cytokines (IFN-γ, IL-6, and TNF-*α*), thereby corroborating NF-*κ*B upregulation (Chen et al. 2015). It is known that IFN-γ may exert a detrimental role in the IAV pathogenesis, leading to an increase in tissue damage; however, it is also true that it is a critical cytokine involved in the regulation of either adaptive or innate immune response necessary for contrasting viral infection(Califano et al. 2018). If saikosaponin A acts by decreasing INF-γ levels, **glycyrrhizin**, from *Glycyrrhiza uralensis* Fisch. (Fabaceae), mediated its antiviral action by stimulating INF-γ release from T cells in IAV-infected (H2N2) mice (10 mg/kg/day, intraperitoneally administrated) (Utsunomiya et al. 1997). However, these data conflict with more recent in vitro investigations showing the anti-inflammatory activity of this compound by decreasing pro-inflammatory cytokine levels. It was indeed demonstrated that glycyrrhizin (25, 50, and 100 µg/mL) anti-influenza activity was related to the inhibition of CXCL_10_, CCL_5_, and IL-6 production and the reduction of lung reactive oxygen species generation with the consequent avoidance of p38, JNK, and NF-*κ*B activation (Michaelis et al. 2011) without interfering with viral replication (Michaelis et al. 2010). The anti-inflammatory activity also seems related to the anti-IAV action of **polyphylla saponin I,** from *Paris polyphylla* var. *yunnanensis* (Franch.) Hand.-Mazz. (Melanthiaceae) (Man et al. 2009). In fact, the oral administration of this compound to IAV-infected mice (5 and 10 mg/kg/day) significantly improved lung tissue pathologic histology and decreased the mortality index. As demonstrated by in vitro investigation, polyphylla saponin I also interfere with viral replication; however, the effective mechanism of action needs to be discovered (Pu et al. 2015). Another oleanane-type triterpene saponin, **chikusetsusaponin IVa**, from *Alternanthera philoxeroides* (Mart.) Griseb (Amaranthaceae), did not show an anti-viral activity against IAV but has in vitro proved to be effective on other enveloped viruses like HSV-1, HSV-2, HCMV, measles virus, and mumps virus (CC_50_/IC_50_ of 29, 30, 73, 25, and 25, respectively). Chikusetsusaponin IVa seemed to act damaging virus envelope leading to a reduction of virus infections. Specifically, this saponin did not inhibit viral attachment and penetration into the host cells or viral synthesis, as it acted by inactivating the progeny viruses released from infected cells, thereby reducing the viral load on uninfected cells (Rattanathongkom et al. 2009). Corroborating in vitro data come in vivo investigation demonstrating that 20 μL containing 0.1 or 0.2 mg of chikusetsusaponin IVa administered intravaginally to HSV-2 -infected BALB/c mice from 3 days before HSV-2 infection to 7 days after infection resulted in dose-dependent protection by increasing survival rate and reducing herpetic lesions severity (Rattanathongkom et al. 2009). Apart from oleanane-type triterpenoid saponins, also dammarane-type triterpenes saponins showed anti-influenza activity. **Ginsenosides** from the American ginseng, *Panax quinquefolius* L. (Araliaceae), are known to have a potent anti-inflammatory activity exerted by lowering the production of pro-inflammatory enzymes such as COX2 and iNOS thanks to the down-regulation of the NF-*κ*B signaling pathway. The ginsenoside Rb1 also reduced the release of TNF-α from macrophages through the inhibition of NF-*κ*B (Kim et al. 2017) and was found to carry out an anti-IAV activity (in mice infected with 10^3^EID_50_ of H1N1 pre-incubated with 1 and 2 mg/mL Rb1) by interfering with the viral HA leading to the attachment avoidance to the α2-3’ salicylic acid receptor of the host cell. It was also demonstrated that the entity of the interaction between ginsenosides and HA is highly related to the number of sugar moieties attached (Dong et al. 2017). This result corroborates those from previous investigations, indicating that sugar motifs and the hydroxyl group number regulated the antioxidant activity of ginsenosides (Zhao et al. 2009b). Ginsenoside Rb1 also exerted an anti-EV71 activity using in vitro (IC_50_ = 0.15 μM) and in vivo (5, 10, and 20 mg/kg/day, intraperitoneally administrated) models not only by increasing the humoral immune response but also by inhibiting the EV71-induced viral protein-2, which is the main EV71 virulence factor for its entrance into the host cells (Kang et al. 2021b). Furthermore, ginsenosides derivatives (Rg6 and Rgx365) incorporated into PEGylated nanoparticle albumin-bound to promote and prolong their bioactivity had been shown as potential molecules for alleviating the inflammation in SARS-CoV-2 ICU patients, thereby reducing the cytokine storm and coagulation. Specifically, it was demonstrated that the formulation might suppress histone H4 elevation and the consequent cytokine storm via down-regulating the NF-*κ*B signaling pathway (Park et al. 2021). Another glycosylated triterpenoid saponin from *Platycodon grandiflorum* (Campanulaceae), the **platycodin D**, demonstrated to interfere with SARS-CoV-2 infection by preventing its entry into the host cells through an alteration of its membrane cholesterol distribution. This activity is related to the molecule structure since it and cholesterol possess a similar size and hydrophobicity; the major differences arise from the presence of an additional elaborate sugar moiety in platycodin D, which cholesterol lacks and that is highly hydrophilic for the presence of sugar moiety hydroxyl groups. This led to the observation that, while platycodin D is similar to cholesterol within the lipid bilayer, outside, the molecule is profoundly different, resulting in a physical hindrance formed by the sugar tail extending out of the membrane. Thanks to this mechanism of action, platycodin D provided a possible SARS-CoV-2 infection therapeutic strategy (Kim et al. 2021). As well as Ginsenoside Rb1, **anemoside B4**, a natural saponin isolated from *Pulsatilla chinensis* (Bunge) Regel (Ranunculaceae). *P. chinensis* roots, exerted an anti-EV71 by regulating the host inflammatory response, as demonstrated in vitro (IC_50_ = 24.95 ± 0.05 μM) and in vivo on infected mice (200 mg/kg/day, intraperitoneally administrated)*.* In particular, the molecules might regulate the Hippo pathway leading to the yes-associated protein phosphorylation and inactivation. The abrogation of YAP/TAZ inhibitory effect on TANK-binding kinase 1 (TBK1) determines the activation of INF-I genes and so the inhibition of EV-71 replication (Kang et al. 2021a). Saponins also showed anti-HBV action. Specifically, **Asiaticoside**, from *Hydrocotyle sibthorpioides* Lam. (Araliaceae), exerted its antiviral activity by suppressing in vitro the level of HBsAg (IC_50_ = 56.9 μM at 7 day and 52.1 μM at 14 day) and HBeAg (IC_50_ = 84.2 μM at 7 day and 67.8 μM at 14 day), extracellular viral DNA, and intracellular cccDNA. In particular, this saponin seemed to markedly reduce the transcription and replication of viral DNA through the activity inhibition of s1, s2, and X genes promoters (Huang et al. 2013b). These HBV promoters may operate as molecular switches, affecting gene activity; deletion of a 'switch' can additionally impair transcription and translation of the HBV gene, resulting in an inhibition of viral replication overall (Pang et al. 2010a, b). HBV inhibition replication was further confirmed in DHBV infected ducks, where a reduction in HBsAg, HBeAg, and viral DNA was also observed. In addition, the in vivo investigation on infected ducks demonstrated that, when compared to lamivudine used as control, asiaticoside (10 and 20 mg/kg/day, intragastrically administrated) was more efficacious in inhibiting HBsAg, HBeAg, and viral DNA rebound, indicating the long-duration effect of this natural molecule. Noteworthy is the effect on the liver as asiaticoside significantly lowered ALT/AST levels, suggesting that, close to its antiviral activity, there was also an improvement in serum biochemistry and hepatocellular architecture (Huang et al. 2013b). Likewise, the triterpenoid saponin **2α,3β,19α-trihydroxyurs-12-en-28-oic acid β-D-glucopyranosyl ester**, from the Tibetan herb *Potentilla anserina* L. (Rosaceae), revealed the ability to reduce in vitro HBsAg (IC_50_ = 57.67 µg/mL) and HBeAg (IC_50_ = 30.05 µg/mL). Moreover, it inhibited HBV at IC_50_ value 19.45 µg/mL. These preliminary experiments were the basis for in vivo experiments performed on Pekin ducklings. More precisely, the compound tested via oral administration at the doses 0.2 and 0.1 g/kg for 5 days inhibited virus DNA replication at 30.30% and 22.16% compared to the control. In addition, the treatment with the molecule at 0.2 g/kg for 10 days significantly reduced virus DNA replication at 58.48% (Zhao et al. 2008).

Until now, the antiviral effect was on envelope viruses, but saponins also showed activity on viruses without envelopes. **Astragaloside IV**, a cycloartane-type triterpene saponin from the roots of *Astragalus membranaceus* (Fish.) Bunge (Fabaceae), showed promising activity in myocarditis induced by CVB3. Preliminary experiments on primarily cultured myocardial cells revealed that astragaloside IV (1 and 5 µg/mL) has an antiviral effect. Subsequent experiments were performed in vivo on CVB3 infected-BALB/c mice (60 and 120 mg/kg, intraperitoneally administrated), showing that astragaloside IV induced a significant decrease in heart necrosis and mononuclear cell infiltration. Moreover, an increase in interferon-γ mRNA expression and a significant decrease in heart weight/body weight ratio (HW/BW) were detected. Finally, a serum pharmacological experiment was performed using the diluted serum of Sprague–Dawley rats, previously treated with 100 mg/kg twice a day for 3 days of astragaloside IV. The blood, taken 1 h after the last dose, induced a decrease in virus titers in primarily cultured myocardial cells. This saponin seemed to act by increasing the expression of INF-γ mRNA and the levels of INF-γ, a cytokine known to lower viral replication or promote the apoptosis of infected cells. These results make astragaloside IV a potential molecule usable for viral myocarditis (Zhang et al. 2006a).

### Flavonoids

Flavonoids are specialized metabolites highly distributed in the plant kingdom, and, up to now, more than 600 varieties have been structurally identified. These compounds are characterized by a flavan nucleus consisting of a skeleton of 15 carbon units forming two benzene rings connected via a pyrene ring. Based on the different chemical substituents in the flavan nucleus, it is possible to distinguish several classes of flavonoids known for their broad spectrum of healthy activities such as antioxidant, anti-inflammatory, antiviral, anticancer, antibacterial, and neuroprotective activity (Dias et al. 2021). This section reviewed the knowledge about the antiviral activities of flavonoids.

#### Flavones

Flavones, the major class in the flavonoid family, are compounds whit a double bond in the flavonoid skeleton between C-2 and C-3, oxidized at the C-4 and without substituent at the C-3 positions. These active molecules act by different mechanisms of action against several viral strains. Specifically, *Scutellaria baicalensis* Georgi (Lamiaceae) is a specie rich in secondary metabolites with promising antiviral activity, especially against IAV infections, such as baicalein, isoscutellarein, and oroxylin A. **Baicalein** (5,6,7-trihydroxyflavone) demonstrated anti-influenza activity both in vivo and in vitro (Chen et al. 2011; Xu et al. 2010), even if it seemed to be related to its main metabolite **baicalin**. Bioavailability tests were therefore carried out to see the concentration of the active metabolite baicalin in the bloodstream following the oral administration of baicalein or baicalin as such. After baicalein oral administration, baicalin reached the maximum level by 2.5 h; on the contrary, after oral administration of baicalin, 10 h were required to reach the maximum levels, which, however, are lower than that obtained after baicalein administration. Baicalein's assumption is then more effective than baicalin, and this is due to the glucoside moiety present in the structure of baicalin which makes it difficult to be absorbed. In fact, after oral administration, baicalin was first converted by sugar removal into baicalein via the intestinal *β-*glucuronidase; hence baicalein was metabolized into the liver to form different metabolites, of which the main one is the active baicalin (Xu et al. 2010). The anti-IAV (H1N1) activity of baicalin was related to the induction of IFN-*γ* release from CD4^+^ and CD8^+^ and natural killer (NK) cells and as a consequence of JAK/STAT-signaling pathway activation. (Chu et al. 2015). The activation of this last signaling pathway by baicalin seemed to be also related to its capacity in modulating the function of NS1 protein, encoded by IAV and known to antagonize cellular antiviral responses by lowering IFN induction and increasing the PI3K/Akt signaling pathway (Nayak et al. 2014). Another mechanism by which baicalin might exert its antiviral activity is related to the modulation of micro-RNAs (mi-RNAs), a class of little non-coding RNA molecules having an important role in blocking the translation or promoting the degradation of mRNAs. It was seen that host microRNAs are highly implicated in adaptive and innate immune reactions and host anti-pathogenic reactions, mainly acting by regulating the host immune system’s vital components. During IAV infection, it is possible to observe a dysregulation of microRNA profiles (for example, miR-146a and miR-155), down-regulation of type I IFN production, and the consequent inactivation of the JAK/STAT signaling pathway. In vivo investigations demonstrated that baicalin acted via suppressing miR-146a with the consequent activation of type I INF response (Li and Wang 2019). With an opposite mechanism of action, baicalin reacted to the lung infection caused by the respiratory syncytial virus (RSV). Specifically, oral administration of baicalin (50, 100, 200 mg/kg/day) resulted in a marked reduction of CD4 and CD8 T lymphocytes and macrophage infiltration in the lung tissues of infected mice, lowering inflammation and viral load. This last effect was most pronounced when baicalin was administered at 100 and 200 mg/kg/day (Shi et al. 2016). Either IAV or RSV viruses are part of the enveloped viruses class; however, baicalin also effectively contrasts the diseases caused by RV, a non-enveloped virus responsible for causing gastroenteritis in children. RV infection determines a reduction in glucose uptake by host cells; baicalin seemed to reverse this condition, possibly through the restoration of sodium-glucose transporter involved in glucose and sodium ions absorption, thereby restoring water-salt balance. This may also account for reducing diarrhoea observed in infected mice after baicalin oral administration (0.15 and 0.30 mg/g). Further, baicalin also avoided gluconeogenesis, contrasting RV ability in enhancing the activity of two rate-limiting enzymes G-6-Pase, and phosphoenolpyruvate carboxykinase and exerting a down-regulation against p-JNK with a consequence up-regulation of pyruvate dehydrogenase kinase 1, Akt, and SIK2 and the inhibition of CBP-CREEB-TORC2 complex formation. These actions not only regulated gluconeogenesis and prevented the RV’s ability to divert gluconeogenesis for saccharide synthesis from non-saccharide substances but also inhibited RV replication in host cells (Song et al. 2021). Similarly to baicalein, also **isoscutellarein** (5,7,8,4'-tetrahydroxyflavone) and its derivative **isoscutellarein 8-methylether**, demonstrated an anti-IAV (A/PR/8/34) activity both in vivo and in vitro (Nagai et al. 1992, 1995). However, contrarily to baicalin, the isoscutellarein derivative might act directly on the virus’s early-stage infection cycle by avoiding the fusion between the endosome/lisosome membrane and the viral envelope (Nagai et al. 1995). **Oroxylin A**, an *O*-methylated flavone, on the other hand, appeared to act with a double mechanism since it directly affected the virus by inhibiting M1 gene transcription and protein synthesis, which is essential for the integrity of IAV, and NA, avoiding the virus diffusion, and indirectly by enhancing the host antiviral defence through the promotion of IFN secretion, especially that of INF-*γ* and INF-*β.* These effects are in vivo corroborated since the oral administration of oroxylin A (25, 50, and 100 mg/kg/day) reduced the body weight loss, increased the survival rate, and ameliorated the pathological changes in the lungs induced by viral infection (Jin et al. 2018). Oroxylin A (intraperitoneally administrated to mice at 10 mg/kg/day) was also active against the non-enveloped RNA virus CVB3, on which it acted by decreasing serum inflammatory cytokine levels and the severity of histopathological lesions in infected mice. Finally, this molecule prevented CVB3-induced cytotoxicity by avoiding eIF2α phosphorylation in response to endoplasmic reticulum stress (Kwon et al. 2016). eIF2α phosphorylation is indeed linked to the formation of intracellular stress granules and macroautophagy, leading to cell death (Bezu et al. 2018).

Flavones have also been reported to exert a protective role again HBV infection. **Nobiletin**, a polymethoxyflavone synthesized in citrus fruit peel (Rutaceae), possessed significant protective properties against the liver and also a promising antiviral activity against HBV either in vivo (15 mg/kg once two days via oral administration) or in vitro (7.5 15, 30, and 60 μM). It acted by suppressing HBsAg secretion and eliminating HBV core DNA. As in vivo demonstrated, this anti-HBV activity was enhanced by the combination with entecavir (nobiletin 15 mg/kg + entecavir 0.02 mg/kg), an approved drug administered during HBV infection, which only reduced HBV DNA levels without reducing HBsAg (Hu et al. 2020). In the same way, **swertisin**, extracted from *Iris tectorum* Maxim rhizomes, exerted in vivo anti-HBV activity (5 mg/kg every other day via intraperitoneal administration) by suppressing HBsAg and HBeAg secretion and eliminating intracellular HBV DNA. Also, in this case, the antiviral activity of swertisin is increased when used in synergy with entecavir (swertisin 5 mg/kg + entecavir 0.03 mg/kg) (Xu et al. 2020). Another flavone that has in vivo demonstrated anti-HBV activity is **luteolin** (20 mg/kg/day, intraperitoneal administration), a 3′,4′,5,7-tetrahydroxy flavone naturally occurring as a glycosylated form and found in several types of plants, including vegetables, spices, and medicinal herbs. For this active metabolite, the mechanism underlying the reduction of HBV antigens and HBV DNA replication was investigated. Specifically, luteolin prevented the hepatocyte nuclear transcription factor-4α from binding to the preC/C promoter, inhibiting its expression. The hepatocyte factor is indeed an important transcription factor that plays a pivotal role in regulating HBV transcription and replication by binding DNA as homodimers. Its suppression induced by luteolin seemed related to the activation of extracellular regulated kinase since its inhibition attenuated luteolin anti-HBV activity (Bai et al. 2016). Luteolin also inhibited in vivo (100 mg/kg/day, oral administration) the replication of DENV-2 by obstructing its later lifecycle stage. An important step in the infectious virion's production that occurs before exiting the infected cell occurs in the trans-Golgi network, where the prM protein is broken down by the host proprotein convertase furin protease. This led to a membrane-anchored M-stump and a 'pr' peptide that stays bound to the virus particle until it is secreted. Luteolin was found to inhibit the dengue viral lifecycle by inhibiting, in an uncompetitive mode, the host proprotein convertase furin protease, thereby obtaining an incorrect breaking of prM protein. In this way, the virus's maturation processes were disrupted, producing less mature virus particles and abrogating viral replication (Peng et al. 2017). The antiviral effect of this flavone was also in vivo investigated against non-enveloped viruses like human enterovirus A71, and it was seen that luteolin (2 and 10 mg/kg/day, intraperitoneal administration), as well as **apigenin** (10 and 50 mg/kg/day, intraperitoneal administration), reduced either EV71 RNA or protein synthesis but with different mechanisms of action (Dai et al. 2019). Specifically, luteolin seemed to target the EV71 post-attachment stage (Xu et al. 2014), while apigenin disrupted the association between the viral RNA and the trans-acting factor and modulated the cellular JNK signaling pathway (Lv et al. 2014a).

As well as nobiletin, from the *Citrus* genus comes the polymethoxylated flavone **tangeretin,** which showed potential antiviral activity against the human respiratory syncytial virus (RSV). This virus is characterized by determining a persistent activation of NF-κB, which results in an excessive gene expression of pro-inflammatory cytokines. Tangeretin was demonstrated to inhibit RSV replication and suppress the viral-induced inflammation in infected mice (25, 50, or 100 mg/kg/day, intragastrically administrated), probably by preventing NF- *κ*B activation resulting in reduced levels of IL-1*β* secretion. However, this molecule did not decrease the mRNA expression and secretion of pro-inflammatory cytokines like INF-γ, IL-4, and IL17a, indicating that tangeretin might modulate RSV inflammatory response by regulating innate but not adaptive immunity (Xu et al. 2015).

#### Flavonols

Flavonols are the most common flavonoids and are particularly ubiquitous in plant food. Also known as 3-hydroxyflavones, these molecules possess a characteristic hydroxyl group at position 3, a double bond between positions 2 and 3, and a ketone group at position 4 of the C ring. Flavonols are reported as compounds exerting the most diverse and interesting biofunctions; quercetin and its derivatives are the most investigated for their antiviral activities. Early in vivo investigations demonstrated that **quercetin** protected mice from Mengo virus infection (Güttner et al. 1982) and that its activity increased when combined with INF-I (quercetin 20 and 10 mg/kg oral + MuIFN-α/β 500 IU parenteral) (Veckenstedt et al. 1987). In the same way, quercetin (12.5 mg/kg/day via oral gavage) protected mice against IV infection (Davis et al. 2008) since it could prevent the early stage of influenza infection by inhibiting the two principal antiviral targets of licensed anti-IAV drugs, HA and NA. Hence, quercetin prevented IAV (H1N1, H1N1, H3N2) infection by avoiding virus entry into host cells through viral HA protein inhibition and the first stage of viral replication linking the active site of viral NA (Liu et al. 2016; Wu et al. 2015). In vivo investigation on infected mice demonstrated indeed that quercetin exerted a dose-dependent (240, 480, and 960 mg/kg twice daily) viral inhibition rate, while the improvement of lung index and the survival rate at 960 mg/kg/die was comparable to that induced by zanamvir at 480 mg/kg/day (Liu et al. 2016). Quercetin was also found to limit the viral replication and symptoms associated with rhinovirus infection. Specifically, this flavonoid reduced either positive or negative strand viral RNA thanks to the lowered cleavage of eIFG4II and the viral capsid protein VP2 reduction. Thus, quercetin was assumed to prevent the processing of the initial polypeptide required for viral RNA polymerase elaboration and eIFG4II cleavage, blocking all downstream stages of RV replication. It was also observed that this flavonol increased eIF2α phosphorylation and, consequently, the host's innate immune responses since the host normally activates this factor to limit viral replication. These hypotheses were confirmed in vivo since quercetin administration to mice (0.2 mg of quercetin daily for 1 or 4 days) prevented not only viral replication but also reduced RV-induced pro-inflammatory chemokine and cytokine expression and airway hyperresponsiveness related to viral infection (Ganesan et al. 2012). Corroborating these data are those from another in vivo investigation in which quercetin, daily administrated with diet (0.1% quercetin providing 100 mg/kg of quercetin) for 10 days, enhanced RV clearance in infected mouse models with chronic obstructive pulmonary disease (Farazuddin et al. 2018). Likewise, the same inhibition in viral replication was demonstrated for EVA71 in newborn mice (Dai et al. 2019), while a Phase I Dose Escalation Study conducted on humans showed the potential application of quercetin (250 to 5000 mg/day) for chronic HCV infection treatment (Lu et al. 2016). As well as quercetin, its derivatives like **isorhamnetin**, **isoquercetin**, **quercetin 3-*O*-*β*-d-glucuronide**, and **quercetin 3‐*O*-**$\boldsymbol{\alpha }$**-l-rhamnoside** demonstrated to have anti-influenza activity in both in vitro and in vivo models by decreasing viral replication and reducing inflammation and oxidative stress (Choi et al. 2012; Dayem et al. 2015; Fan et al. 2011; Kim et al. 2010). Specifically, isorhamnetin inhibited the first steps of IV replication by inhibiting NA (Dayem et al. 2015) and also protected from EVA71 infection (Dai et al. 2019). On the other hand, isoquercetin prevented ebolavirus infection by inhibiting viral entry into the host cells, probably affecting the glycoprotein-mediated step (Qiu et al. 2016). The 3-hydroxy derivative of quercetin, **dihydroquercetin**, a flavonoid extracted from *Larix sibirica* wood, showed antiviral activity against either non-enveloped virus, like coxsackievirus B4 or enveloped virus, as IAV; however, the mechanism of action was not discovered (Galochkina et al. 2016; Trofimova et al. 2015). With a greater antiviral effect than quercetin, other 3-hydroxyl group flavonoids **3,2′-dihydroxyflavone** and **3,4′-dihydroxyflavone** acted, demonstrating the importance of the hydroxyl group substitution. These two compounds exerted their anti-influenza effect by inhibiting the HA and NA activity, as also demonstrated by in vivo investigation where a reduced lung viral triter was evidenced after oral administration of 1 mg/kg/day for 5 days to infected mice (Hossain et al. 2014). Other flavonols with potential activity again IAV infection is **kaempferol** (3,5,7-trihydroxy-2-(4-hydroxyphenyl)-4H-1-benzopyran-4-one) from several edible plants (e.g., broccoli, tea, kale, cabbage, endive, beans, leek, strawberries, tomato, and grapes) and plants or botanical products generally used in traditional medicine (e.g., *Tilia* spp., *Ginkgo biloba*, *Moringa oleifera*, *Equisetum* spp., *Sophora japonica*, and propolis). In particular, kaempferol showed antiviral activity against peramivir and oseltamivir-sensitive and resistant influenza viruses both in vitro and in vivo. These active molecules seemed ineffective in preventing absorption or invasion but might act by suppressing the later replication stage (Kai et al. 2014). As previously mentioned, the host cell redox stage plays a pivotal role in viral replication. Kaempferol was able to mitigate ROS production and the formation of MDA, a product of the lipid peroxidation breakdown; hence the inhibition of IAV replication in mice after kaempferol intragastric administration (15 mg/kg) should be related to the restoration of the redox state in mice. In addition to the antioxidant activity, kaempferol attenuated inflammation and pulmonary oedema in infected mice by down-regulating TLR4/MyD88-mediated NF-*κ*B and MAPKs signaling pathway, thereby reducing the release of pro-inflammatory cytokines like TNF-*α*, IL-6, and IL-1*β* (Zhang et al. 2017a). A suppression of cell-autonomous immunity was confirmed in another in vivo study investigating the anti-IAV effect of kaempferol (100 mg/kg, intragastric administration); however, in this case, no antiviral activity was demonstrated since an increase in virus replication was observed (Dong et al. 2014). Hence further investigations are needed to confirm the kaempferol antiviral activity against IAV. This flavonol also demonstrated an effect against EV71 by interfering with viral replication and inhibiting the internal ribosome entry site (IRES) activity leading to a limitation of viral infection (Dai et al. 2019; Tsai et al. 2011). This effect resulted in an increase of survival rate about 88.8% when kampferol was intraperitoneally administered to infected mice at the dosage of 50 mg/kg (Dai et al. 2019). An anti-EV71 effect was also demonstrated for *O*-methylated flavonols as **penduletin** and **chrysosplenetin,** isolated from *Laggera pterodonta* (DC.) Sch.Bip. ex Oliv. leaves (Dai et al. 2019; Zhu et al. 2011). The specific mechanism of action by which these molecules acted need further investigation, but preliminary studies demonstrated that it did not block virus entry nor inhibited viral RNA replication on Vero cells but exerted an antivitral activity with an IC_50_ of 0.17 ± 0.13 and 0.17 ± 0.12 µM for chrysosplenetin and penduletin, respectively (Zhu et al. 2011). Apart from EVA71either penduletin or chrysosplenetin had demonstrated in vitro to be effective inhibitors of a broad spectrum of other human enteroviruses (EV84, EV11, CVB3, CVA10, and CVA16) (Zhu et al. 2011).

Flavonols were also investigated for their anti-HSV activity. **Houttuynoid A**, extracted from *Houttuynia cordata* Thunb., inhibited in vitro viral entry by blocking the fusion between the viral envelope and the host plasma membrane (IC_50_ = 23.50 ± 1.82 μM). Moreover, houttuynoid A (100 µM mixed with 1.0 × 10^7^ PFU HSV-1/F and added to the broken mice skin) inhibited HSV-1 infection in the BALB/c mouse model, reducing the viral loads in the infected skin tissue (Li et al. 2017). Likewise, **myricetin**, a common dietary compound occurring in vegetables, fruits, nuts, berries, tea, and red wine, blocked the viral entry through direct interaction with the viral gD protein expressed on the enveloped, inhibiting adsorption and membrane fusion. Furthermore, myricetin also inhibited the EGFR/PI3K/Akt signaling pathway, which is essential for HSV replication. As a result, there was an anti-HSV activity of myricetin after the intraperitoneal administration of 2.5 or 5 mg/kg/day to infected mice (Li et al. 2020b). Besides this anti-HSV action, it was observed that myricetin reduced influenza virus replication in infected mice, but the mechanism of action needs to be investigated (Yoo et al. 2013).

#### Flavanones

Flavanones are another class of flavonoids comprising $\sim$ 350 aglycones and 100 glycosylated forms. The basic 2,3-dihydroflavone structure characterizes them, but they differ from flavones and flavonols in the absence of the C2-C3 double bond, the substitution in C3, and the presence of a chiral atom in C2 (Barreca et al. 2017). They are widely distributed in nature and represent intermediates of the flavonoid biosynthetic pathway. **Hesperidin** (3,5,7-trihydroxyflavanone-7-rhamnoglucoside), the glycosidic metabolite of hesperetin, is the main dietary flavone glycoside found in *Citrus* species and is also known as Vitamin P (Srinivasan et al. 2019). This specialized metabolite is known for its anti-inflammatory activity, possibly also attributable to its antiviral effect against IAV infection. In fact, hesperidin did not inhibit viral replication but acted by reducing pro-inflammatory cytokines via MAPK signaling suppression (Ding et al. 2018). Specifically, hesperidin enhanced the expression and activation of P38 and JNK, improving cell-autonomous immunity. P38 is indeed a critical protein for the antiviral response since its phosphorylation and nucleus translocation was related to many transcription factor activation like NF-κB, AP1, and Jun, and STAT1 phosphorylation, known to be directly involved in the transcription of INF-γ (Ding et al. 2018). This anti-IAV activity was in vivo corroborated as the hesperidin, intragastric administration to infected mice (100 mg/kg/day) and intraperitoneally injected to infected rats, attenuated pulmonary inflammation and pathology by reducing pro-inflammatory cytokines like TNF-*α*, IFN-*α*, and IL-6 (Ding et al. 2018; Dong et al. 2014). Instead of hesperidin, another flavanone from *Citrus* species, **prunin**, appeared to interfere directly with the replication of other enveloped or non-enveloped viruses by disrupting viral protein and RNA synthesis. In either EV71 or HCV, prunin inhibited the viruses’ internal ribosome entry site, which is known to initiate viral RNA translation (Gunaseelan and Wong 2019). In fact, several RNA viruses with positive-strand uncapped genomes use internal ribosome entry site elements to control viral protein synthesis (Martinez-Salas et al. 2018). Prunin anti-EVA71 effect was also demonstrated in vivo (1, 3, and 10 mg/kg/day, intraperitoneally administrated). EVA71 infection provokes the accumulation of neutrophils and macrophages and the secretion of proinflammatory cytokines with muscle tissue destruction. Due to the reduced viral antigen distribution, these muscle tissue damages seemed restricted when prunin was administered (Gunaseelan and Wong 2019). **Pinostrobin,** a natural monohydroxyflavanone deriving from the leaves of *Cajanus cajan* (L.) Millsp. (pigeon pea), also exerted its antiviral activity by directly acting on the virus as demonstrated in vitro (EC_50_ = 22.71 ± 1.72 µg/mL) and in vivo on infected mice (20 and 50 mg/kg/day via oral administration). In this context, important was its anti-HSV activity by preventing virus entering inside the host cell. Pinostrobin targeted the surface of the viral lipid envelope, thereby causing a gradual leakage and breakage of the envelope and HSV inactivation. In this way, virions were unable to infect the host cells (Wu et al. 2011b). The lipid envelope contains several glycoproteins (e.g. gB, gC, gD, etc.) consisting of polypeptides with several proton donors and acceptors and which are necessary for the entry of HSV-1 into the healthy host cells. Looking at the chemical structure of pinostrobin it is possible to observe the presence of methoxyl, hydroxyl, and carbonyl substituents capable of forming hydrogen bonds with the glycoproteins donors and acceptors protons groups. Thus, the hydrogen bonds formed between pinostrobin and virions glycoproteins might determine the desquamation and disruption of the viral envelope (Wu et al. 2011b).

#### Isoflavones

Isoflavones are a subclass of flavonoids mainly produced by the members of Leguminosae; they are characterized by the 3-phenylchromen-4-one backbone principally modified by prenylated and/or glycosides derivatives and *O*-substituents. In plants, the greater isoflavones concentration has been found in soy (*Glycine max* L.), kudzu, and red clover (*Trifolium pratense*). Like the other flavonoids, isoflavones’ antiviral targets were virus envelop glycoproteins, leading to the prevention of viral binding end entry and viral replication, with the consequent inhibition of viral protein translation. An example of abundant isoflavone mainly found in soybeans and soy products is **genistein**, also known as prunetol, which was reported to act as a general tyrosine kinase inhibitor and reported to inhibit viral infection in vitro by preventing viral entry. Further, genistein was evidenced to prevent new influenza virions from being released and spread by inhibiting NA active sites either in vivo (0.4 g/kg/day, orally administered) or in vitro (IC_50_ = 3058.8 ± 218.9 and 2729.6 ± 275.1 µM for A/NWS/33 (H1N1) and A/chicken/Korea/MS96/96 (H9N2), respectively) (Wei et al. 2015). Soy products also contain **formononetin,** which demonstrated in vivo antiviral activity after intraperitoneal injection (10 mg/kg/day) to EV71- infected mice infected with EV71 (Dai et al. 2019). In fact in vitro study showed that this active molecule inhibited EV71 probably through the down-regulation of inflammatory pathways like ERK, p38, JNK, and MAPK, which were upregulated during infection and played a pivotal role in helping viral reproduction (Wang et al. 2015). Another isoflavone, the **calycosin 7-*O*-*β*-d-glucopyranoside**, isolated from *A. membranaceus* var. *mongholicus* (Bunge) P.K.Hsiao, showed promising protection against CVB3-causing myocarditis. It was indeed in vivo demonstrated that the compound (24 mg/kg/day, orally administered) prevented viral replication in the heart and reduced inflammation, resulting in heart protection and increasing infected mice’s survival rate (Zhu et al. 2009).

#### Chalcones

Chalcones (1,3-diphenyl-2-propene-1-one) form another flavonoid family class structurally characterized by two aromatic rings, which should be polyhydroxylated, linked through three carbon *α,β*-unsaturated keton system. To date, chalcones have not been extensively investigated for their potential application as antiviral molecules; however, the few literature data indicated that their antiviral activity depends on the presence of a specific substituent. For instance, the electron-donating group presence in the *p*-position of the aromatic group (CH_3_, NMe_2_, and OCH_3_) showed an important activity against HSV infection (Marinov et al. 2020). On the other hand, the prenylated chalcone **xanthohumol** (3′-(3,3-dimethylallyl)-2′,4′,4-trihydroxy-6′-methoxychalcone), from the female inflorescences of *Humulus lupulus* L. plant (hops), demonstrated a selective inhibition against HIV-1 infection probably by blocking the reverse transcriptase and p24 antigen (Wang et al. 2004). Contrarily, no effective anti-HCV activity was demonstrated; however, its oral administration (1 mL/100 g body weight, divided into 3 single daily doses) to infected *Tupaias* reduced hepatic inflammation, fibrosis, and steatosis by lowering oxidative reaction, regulating apoptosis, and suppressing hepatic stellate cells activity (Yang et al. 2013a).

#### Catechins

Other members of the polyphenol group are catechins structurally characterized by the basic flavan 3-ol nucleus. Structural-activity relationships have demonstrated that the 5’-OH and 3-galloyl groups are essential for their antiviral activity against a broad range of viruses. **Epigallocatechin 3-gallate** is the main catechin found in green tea (*Camelia sinensis*) and is known to be promising in treating and/or preventing infection of DNA, like HBV, and RNA, as HCV, CVB3, and IAV viruses. Specifically, this compound reported robust anti-HBV activity in vivo (50 mg/kg two times a day for 4 weeks) by reducing the relaxed circular DNA (rcDNA) and the HBsAg mRNA (Lai et al. 2018). In contrast, activity against HCV is uncertain; in vitro experiments demonstrated that epigallocatechin 3-gallate might prevent viral attachment to the host cells by interacting with the envelope glycoprotein; at 25 µg/mL of the compound, almost 100% of viral inhibition. However, monotherapy (100 mg/kg twice daily by oral gavage) failed to protect mice from HCV infection, probably due to its lower oral bioavailability (O'Shea et al. 2016). Promising protection against CVB3-induced myocarditis was instead highlighted in both in vitro and in vivo studies. ECCG significantly down-regulated CVB3 replication by inhibiting protein expression levels and the adenovirus receptor, known to be the main viral receptor involved in myocytes infection. However, epigallocatechin 3-gallate failed to reduce the expression of pro-inflammatory cytokines (TNF-α, MCP-1, and IL-6) induced by the viral infection in infected mice (10 mg/kg/day via oral administration) (He et al. 2017). This inability of epigallocatechin 3-gallate to reduce inflammation is inconsistent with studies investigating its potential anti-IAV infection in vivo (10, 20, and 40 mg/kg/day via oral administration) and in vitro (ED_50_ value was 8.71 ± 1.11 nmol/L). In this case, indeed, the natural compound exerted its antiviral action by interrupting the early phase of the viral replication cycle and down-regulating the TLR/NF-κB signaling pathway, thereby reducing the levels of inflammatory cytokines and oxidative stress (Ling et al. 2012; Xu et al. 2017). Another catechin, **gallocatechin 7-gallate**, from *Pithecellobium clypearia* (Jack) Benth. leaves and twigs, also showed an important antiviral effect against IAV-infected mice (30 mg/kg/day, intravenously administrated) by inhibiting the Host-cdc2-like kinase 1, known to be responsible for the phosphorylation of the arginine- and serine-rich factors. These last include SRp20, 9G8, SC35, and SF2/ASF involved in regulating the spliceosome assembly early events, which are essential for mRNA maturation. It was demonstrated that Host-cdc2-like kinase 1 inhibition could inhibit viral replication by more than two orders of magnitudes through the M2 mRNA-impaired splicing. Gallocatechin 7-gallate, by inhibiting Host-cdc2-like kinase 1, down-regulated SF2/ASF and SC35 phosphorylation leading to the impaired synthesis of the IAV proteins M2 and NP. Furthermore, as well as epigallocatechin 3-gallate, gallocatechin 7-gallate also reduced the expression of pro-inflammatory cytokines IL-6, TNF-*α*, and IL-1*β* (Li et al. 2018).

### Phenylpropanoids

Phenylpropanoids, plant phenolic acids characterized by a C_6_-C_3_ carbon skeleton, are derived mainly from cinnamic acid. Cinnamic acid owes its name to *Cinnamomum*, a taxonomic genus belonging to the Lauraceae family, cinnamon, camphor, and related plants. **Chlorogenic acid**, the ester of caffeic acid with quinic acid, has numerous biological properties, including antiviral activity. Influenza A H1N1-infected mice were treated with different doses of chlorogenic acid, including 240, 480, and 960 mg/kg/day, through oral administration, and it was seen that chlorogenic acid improved survival rates from 40% (240 mg/kg/day) to 56% (960 mg/kg/day) (Liu et al. 2016). However, the mechanism of action of chlorogenic acid to inhibit viral infections need to be further explored. Another phenylpropanoid derivative isolated from several dicotyledons, **acteoside**, also known as **verbascoside**, acted through a different mechanism of action against IAV. Its antiviral activity was found to be highly related to the increase in IFN-γ production in mouse T cells dose-dependently (20, 40, 80, and 160 µM), probably thanks to the activation of T-bet and ERK phosphorylation cascade. In regulating IFN-γ gene expression and secretion are involved several signaling factors, such as the transcription factor T-bet and the activation of the MAPK signaling pathway. This last is responsible for ERK activation resulting in a phosphorylation cascade ending with the IFN-γ release. On the other hand, T-bet is a transcription factor belonging to the T-box family member, is expressed in T cells like CD4+ and CD8 + , and was found to be essential for obtaining the maximal transcription of IFN-γ. Acteoside increased IFN-γ levels by a double mechanism, including the activation of T-bet and the increase in the phosphorylation of ERK1/2, probably by activating MAPK. Differently from cytokine stimulation, which normally leads to IFN-γ release and cytotoxicity, selective activation of IFN-γ production of CD4+ and CD8 + cells by the acteoside provides a good opportunity to differentiate between the two main functions of CD3 cells, involving cytokine generation and cytotoxicity, particularly when cytotoxicity can cause normal tissue damage (Song et al. 2016). Acteoside has also been found to have a protective effect against RSV infection since it can reduce RSV replication in vivo (80 mg/kg, intraperitoneally administrated), even if the exact mechanism of action needs to be investigated (Chathuranga et al. 2019). Another phenylpropanoid with antiviral activity is **cinnamaldehyde,** which demonstrated both in vivo (250 μg/mouse/day through nasal inoculation) and in vitro (20–200 μM) anti-influenza activity thanks to its capacity to reduce viral protein synthesis at the post-transcriptional level (Hayashi et al. 2007). The effect of cinnamaldehyde on CVB3 was instead unclear, probably for its rapid conversion to **cinnamic acid**. In vitro experiments reported that cinnamic acid (100–1000 µM) but not cinnamaldehyde (100–1000 µM) reduced the viral titer in the CVB3-infected myocardial cells with low cytotoxicity. On the contrary, cinnamaldehyde but not cinnamic acid (30 mg/kg, intraperitoneally) reduced inflammation and oxidative stress by inhibiting the TLR4-NF-*κ*B signaling pathway in CVB3-induced myocarditis in mice (Ding et al. 2010). Cinnamic acid was also reported to block Zika virus replication, both in vivo (75 or 150 mg/kg) and in vitro, by inhibiting the RNA-dependent RNA polymerase (RdRp) activity (Chen et al. 2021). With promising antiviral activity, there is also **rosmarinic acid**, a phenolic compound found in various Lamiaceae plants, which has been demonstrated to be active against EV71 and Japanese encephalitis virus (JEV). The compound (100 mg/kg) inhibited the early phases of viral infection, like EV71 attachment and entry into the host cells, targeting several cellular receptors such as the scavenger receptor class B member 2 (SCARB2), P-selectin glycoprotein ligand-1 (PSGLI), and heparan sulfate glycosaminoglycan (Hsieh et al. 2020). Furthermore, rosmarinic acid (20 mg/kg, body weight) might also interact with EV71 proteins like VP1 protein, with which SCARB2 normally interact to start the EV71 protein at low pH condition (Lin et al. 2019). Regarding JEV, the compound administration to infected mice (25 mg/kg, intraperitoneally) reduced brain viral replication and the secondary inflammation related to microglial activation. However, the mechanism of action underlying this activity needs to be understood (Swarup et al. 2007).

### Lignans

Lignans are a naturally occurring large group of compounds characterized by a basic scaffold formed by two or more phenylpropanoid monomers like cinnamic acid, propenyl benzene, allyl benzene, and cinnamyl alcohol. When the monomers’ molecular linkage is formed between position β-β’, the compounds are known as “classical lignans”, while the others that do not contain this linkage are designed as “neolignans”. Several lignans have been revealed to have interesting antiviral properties by inhibiting, for example, the transcriptase, integrase, and topoisomerase leading to the inhibition of viral replication.

#### Classical lignans

**Phillyrin**, isolated from the fruits of *Forsythiae suspensa* (Thunb.) Vahl. (Oleaceae), has been reported to possess antiviral activity against IAV infection (A/FM/1/47) in vivo (10 or 20 mg/kg/day, intraperitoneally administrated)*,* decreasing the expression of influenza hemagglutinin (HA) protein and the IL-6 levels in the serum of infected mice. Thus, phillyrin may protect mice against IAV infection by inhibiting viral replication and reducing viral-induced inflammation (Qu et al. 2016). Similarly, **arctigenin** and its glycoside **arctiin**, a phenylpropanoid dibenzylbutyrolactone lignan extracted from the fruits of *Arctium lappa* L. (Asteraceae), acted at the first stage of IAV infection (A/NWS/33, H1N1), inhibiting viral replication and increasing the immune response both in vivo and in vitro even if the specific mechanism of action needs further investigations. In mice, arctiin was immediately metabolized into arctigenin after administration and remained in the blood for 12 h*,* suggesting that arctigenin is the active metabolite responsible for the antiviral activity. It is possible to think that arctigenin interfered with early intracellular stages after virus penetration into the host cells and with the release of progeny viruses as well; the coadministration of arctiin (1 mg/day) and oseltamivir (0.02 or 0.05 mg/day) increased its anti-IAV activity (Hayashi et al. 2010). Arctigenin also demonstrated antiviral activity against JEV infection since its intraperitoneal administration (10 mg/kg) significantly reduces brain viral load and replication, inflammation, and oxidative stress responsible for neuronal death (Swarup et al. 2008). **Diphyllin** is a natural arylnaphthalene lignan belonging to the vacuolar ATPase (V-ATPase) inhibitors class that can intercept virus entry into host cells. V-ATPases are eukaryotic cells’ ubiquitous proton pumps found in the endomembrane system which are responsible for the endosomal acidification essential for virus entry. Hence, the inhibition of V-ATPase prevents viral infection. However, diphyllin is characterized by poor solubility, which limits its application in antiviral treatment; for this reason, it was encapsulated in nanoparticles of poly(ethylene glycol)-block-poly(lactide-coglycolide) (PEG-PLGA) and intravenously injected to mice (diphyllin nanoparticles contained 10 µg of diphyllin) for 1 or 3 days. Under this formulation it was seen an increase of diphyllin anti-IAV activity (Hu et al. 2018). A derivative of diphyllin, the **6-deoxyglucose-diphyllin**, isolated from *Justicia gendarussa* Burm.f (Acanthaceae), exhibited antiviral activity against Zika virus (ZIKV) both in vivo and in vitro. As for diphyllin, mechanistic studies showed that 6-deoxyglucose-diphyllin blocked ZIKV infection by inhibiting the acidification of endosomal/lysosomal compartments in target cells, which is critical for entering viral particles into the target cells (Martinez-Lopez et al. 2019). Another lignan **schisandrin A**, isolated from *Schisandra chinensis* (Turcz.) Baill. (Schisandraceae), was reported as a potential antiviral agent against DENV in vitro (EC_50_ = 28.1 ± 0.42 μM on Huh-7 cells) and in vivo (5 and 10 mg/kg)*.* Schisandrin A inhibited DENV RNA replication and protein synthesis and increased the production of IFN*-α* protein and STAT1/2 phosphorylation, which are involved in IFN-mediated antiviral responses, suggesting that schisandrin A could exert its virucidal activity by inducing the antiviral IFN response (Yu et al. 2017b).

#### Neolignans

As well as methyl helicterate, from the Meliaceae family comes another anti-HV promising molecule, **silvestrol**, a neolignan characterized by cyclopenta[b]benzofuran moiety isolated from *Aglaia foveolata* Pannell. This compound inhibited HEV viral replication in different HEV experimental model systems like humanized HEV-infected mice at 0.3 mg/kg and acted additively to ribavirin (RBV). Specifically, silvestrol would target the host factor eIF4A during cap-dependent translation initiation. In this way, silvestrol blocked the mRNA translation machinery, which is known to be formed by eIF4A, eIF4G, and eIF4E. In fact, it was demonstrated that eIF4E-binding protein 1 (4E-BP1) was implied in the translation complex's negative regulation, corroborating the importance of eIF4A and eIF4E in sustaining HEV replication (Todt et al. 2018).

#### Flavolignans

**Silymarin** is a flavolignans mixture extracted from the milk thistle of *Silybum marianum* (L.) Gaertner (Asteraceae), used since ancient times in the therapy of liver diseases, and composed of five major compounds: silybinin, isosilybinin, silychristin, silydianin, and taxifoline. The major active component of silymarin consists of the two stereoisomers silybin A and silybin B (ratio 1:1), also known as **silibinin**. Silymarin has extensively known for its hepatoprotective effect attributable to its anti-inflammatory, antioxidant, and immunomodulatory effects, which led this molecule to be a candidate for the treatment of viral hepatitis. In fact, the administration of silymarin to 67 patients with acute HAV, HCV, HBV, cytomegalovirus, enteric, and dengue virus decreased ALT serum levels, demonstrating that silymarin was effective in patients with acute hepatitis (Jaffri et al. 2007). However, despite its good potential therapeutical activity, silymarin has poor solubility, affecting its bioavailability in vivo. Thus, the hydrophilized silibinin, Legalon^®^ SIL was developed by the pharmaceutical company Rottapharm Madaus (Monza, Italy) to treat hepatic intoxication by *Amanita phalloides* mushrooms and also for the prevention of recurrent hepatitis C in liver transplant patients (Guedj et al. 2012). Several works demonstrated the anti-HVC activity of this drug, but it is important to consider possible mutations that make resistant HCV to SIL. The analysis of viral genomes revealed that SIL targeted the interaction between NS4B and NS3/4A, which is responsible for membrane alteration and the formation of functional HCV replication sites. These results suggested that the antiviral activity of SIL could be partially mediated by inhibiting the HCV replication site formation, interfering whit the interaction between NS4B and NS3/4A. A mutation in the *C*-terminal region of NS4B significantly reduced HCV sensitivity to Legalon^®^ SIL, conferring resistance to the treatment (Esser-Nobis et al. 2013). In a study on HCV-infected uPA-SCID chimeric mice with humanized livers, SIL was administered for 14 days (61.5, 265, and 469 mg/kg, intravenous administration), demonstrating that this molecule might block viral production and increase anti-inflammatory and antiproliferative gene expression in human hepatocytes of treated mice (Deb Roy et al. 2016). These effects were further validated by clinical trial since Legalon^®^ SIL administration (10, 15, or 20 mg/kg/day) resulted in a progressive HCV-RNA reduction and a lack of viral breakthrough without adverse effects during the period of iv- Legalon^®^ SIL monotherapy, even if monotherapy did not avoid reinfection (Bárcena et al. 2013; Guedj et al. 2012). This last result was in contrast with that obtained by a single-centre, prospective, randomized, parallel-group, double-blind, placebo-controlled, phase 2 trial where, after the intravenous treatment with Legalon^®^ SIL (20 mg/kg/day), viral load decreased 16 days after the end of treatment and was similar to baseline (Rendina et al. 2014). The arrest of HCV infection shown after Legalon^®^ SIL intravenous administration (20 mg/kg/day) is associated whit a decrease in the severity of liver disease (Canini et al. 2015). Further, short-term and high-dose silibinin infusion (1400 mg/day for two consecutive days) determined complete viral suppression in patients with minimal residual viremia during standard therapy with interferon and RBV (Biermer et al. 2012). The antiviral activity of Legalon^®^ SIL was also associated with its immunomodulatory effects, as demonstrated in 12 liver transplant patients with recurrence of HCV. In this case, a decrease in HCV viral load was observed in association with changes in the levels of pDC and mDC represented by an increase of pDC/mDC ratio, while no effects were observed in Treg frequency or programmed death (PD)-1 expression by Treg, demonstrating that SIL exerted antiviral activity through its immunomodulatory effects, but did not affect PD-1/PD-L1 pathway involved in the persistence of HCV infection. However, several correlations between DC/Treg markers and clinical parameters were detected, suggesting that Legalon^®^ SIL (20 mg/kg/day, intravenous administrated for 14 days) could ameliorate the clinical condition of liver transplant patients (Castellaneta et al. 2016). The fact that daily intravenous silibinin was safe and showed significant antiviral activity could suggest its longer treatment in clinical trials to prevent hepatitis C recurrence, especially in patients with liver transplants (Mariño et al. 2013). The efficacy of Legalon^®^ SIL against HCV in patients non-responders to a full dose of peginterferon/RBV (PegIFN/RBV) combination therapy was also demonstrated. In this case it was seen that intravenous silibinin therapy decreased the viral load in a dose-dependent manner, but its effect did not improve the response to interferon because the antiviral activity was not maintained after the end of the infusion period. However, Legalon^®^ SIL was well tolerated, with no serious adverse effects observed, confirming it as a safe antiviral agent against HCV in non-responders (Ferenci et al. 2008). These results agree with that of another clinical trial confirming that intravenous Legalon^®^ SIL could be considered a “rescue treatment” for patients on treatment and non-responding to standard therapy (Rutter et al. 2011). Furthermore, combination therapy of pegylated interferon RBV and silibinin in non-responder and post-liver transplantation patients reduced the viral load that became undetectable in week 6, resulting in a sustained virological response 24 weeks after the end of therapy. Hence, combination therapy with silibinin, pegIFN, and RBV (20 mg/kg/day, 135 μg/week, and 600 mg/day, respectively), might be effective for treating non-responder patients reinfected with HCV and liver transplanted (Knapstein et al. 2014). Intravenous Legalon^®^ SIL was also demonstrated to reduce viral load and increase CD4+ cell counts in HIV/HCV co-infected patients non-responding to standard double therapy with peginterferonα-2a and RBV (Payer et al. 2010). These results were confirmed in two pilot studies where intravenous silibinin was administered to HIV/HCV co-infected patients with advanced liver fibrosis and non-responder to peginterferon-RBV therapy. In fact, a lead-in therapy with silibinin reduced HCV RNA and 63% of patients showed a sustained virological response at week 12 after the end of treatment (Braun et al. 2015). Despite the observed effectiveness of intravenous Legalon^®^ SIL, an oral formulation of silibinin encapsulated in nanoparticles (< 200 nm) was developed to enhance its solubility and bioavailability. This formulation (50 μM) exhibited in vitro anti HCV activity in human hepatocytes, especially during the virus's intercellular spread, and a potent antioxidant activity that could eliminate HCV-induced oxidative stress. Furthermore, oral administration of silibinin nanoparticles in rodents (5 or 10 mg/kg) produced no apparent adverse effects but improved the bioavailability and liver distribution compared to free silibinin, supporting the potential of these nanoparticles (Liu et al. 2017). Another formulation is that of silybin phospholipids and vitamin E complex (SPV complex, 47 mg silybin and 15 mg vitamin E), which demonstrated hepatoprotective and anti-inflammatory effects in patients with chronic HCV infection since an improvement in viral load and hepatic indices and a persistent reduction of serum ALT, AST, and IL-6 levels was observed (Falasca et al. 2008). It is also possible to administrate SPV complex (silybin 94 mg + vitamin E 30 mg + phospholipids 194 mg in pills for 12 months) in HCV patients treated with pegIFN and RBV standard therapy. Results showed that supplementation of silybin not only increased the effect of pegIFN and RBV but also improved the quality of life of HCV patients, ameliorating the workability and reducing depression and anxiety, compared to no silybin treated group (Malaguarnera et al. 2016).

Apart from Legalon^®^ SIL, also silymarin was investigated for its anti-HCV activity in clinical studies. In particular, silymarin administration (420 or 1050 mg/day) to patients with chronic HCV and decompensated cirrhosis resulted in reduced ALT and AST levels without adverse effects, even at the highest dose (Fathalah et al. 2017). This molecule was also active in patients with HCV non-responding to prior peginterferon/RBV since silymarin administration was associated with a reduced progression of hepatic fibrosis to cirrhosis (Freedman et al. 2011). However, pharmacokinetics studies demonstrate that due to low bioavailability, oral doses of silymarin higher than 2.1 g/day doses or longer treatment may be necessary to achieve the concentrations for antiviral effects since doses below 700 mg did not show a significant reduction of serum transaminase and HCV RNA titer (Hawke et al. 2010). In fact, the administration of 3 capsules daily of silymarin (125 mg) did not affect HCV RNA titers, serum ALT, and serum and ultrasound markers for hepatic fibrosis, suggesting further studies with a longer treatment period or higher dose (Tanamly et al. 2004).

### Tannins

Tannins are polyphenolic molecules of natural origin normally found in all plant parts (stems, roots, leaves, seeds, and fruits), which explains their presence in numerous natural sources. They are known for many physiological activities related to their ability to bind proteins, and thanks to that, tannins exert antiviral activity against a broad spectrum of viruses. **Chebulagic acid, geraniin,** and **punicalagin** are hydrolyzable tannins that could inhibit the EV71 either in vivo or in vitro*,* probably by counteracting the viral absorption/penetration since a reduction in EV71 replication and the viral cytopathic effect was observed (Yang et al. 2012b, c, 2013b). Further, the intranasal administration of both chebulagic acid and punicalagin (50 mg/kg of each) to RSV-infected mice was demonstrated to reduce viral lung loads and alleviate viral-induced lung lesions by suppressing COX-2, iNOS, and PGE2 protein expressions and down-regulating MAPK and IKK-NF-*κ*B signaling pathway (Xie et al. 2016). Geraniin was instead investigated for its anti-DENV activity through in vitro and in vivo models about its ability to bind viral protein*.* In fact, when geraniin was intravenously administrated to infected mice (131.30 μM prepared in 100 μl PBS), it rapidly bound the circulating DENV-2 viral protein, thereby preventing the infection and the virus-induced splenomegaly (Abdul Ahmad et al. 2019). Specifically, using in vitro models (IC_50_ = 1.75 μM), it was seen that geraniin directly linked the domain III region of viral E protein with consequent inhibition of DENV viral entry and replication (Abdul Ahmad et al. 2017). Another tannin, **eugeniin**, showed interesting anti-HSV-1 related to its capacity to inhibit the HSV-1 DNA polymerase activity non-competitively in the vicinity of the phosphonoacetic acid (a licensed drug for treating HSV1 infection) binding site, leading to the prevention of viral replication. This antiviral activity was confirmed by the virus yield reduction in infected mice’s skin and brain, thus ameliorating herpetic symptoms (6 or 50 mg/kg of eugeniin, administrated orally or intraperitoneally) (Kurokawa et al. 2001). With a different mechanism of action, acted **corilagin**, a hydrolyzable tannin able to ameliorate brain inflammatory impairments caused by HSV1 in infected mice (0.4 mg/each mouse, administered intragastrically). Specifically, corilagin acted by inhibiting the TIRAP/MyD88-TRAF6 signaling pathway, a molecular pattern responsible for initiating inflammation and innate immune response and which usually begins after the activation of TLR2. Inflammation was further reduced by inhibiting NEMO, P38, p-P38, NF-*κ*B, TNF-*α*, and IL-6 expression, thus reducing the brain-inflammatory lesions related to HSV1-induced encephalitis (Guo et al. 2015). Corilagin was also active against HCV, as demonstrated in infected chimeric mice (1 mM/day, administrated orally). It might act by inhibiting HCV replication kay enzymes like NS3 protease and NS5B RNA-dependent-RNA-polymerase and suppressing mRNA levels of NOX4 and TGF-*β* with a consequent reduction of oxidative stress (Reddy et al. 2018). As evidenced by dietary supplements to HBeAg-Transgenic mice with chronic HBV infection, protection against viral hepatitis was also provided by **ellagic acid** (5 mg/kg body weight), a natural phenolic molecule contained and released by ellagitannins. Although the function of secretory HBeAg in the viral life cycle is neither known nor necessary for infection or replication processes, host immune tolerance induced by it represents a viral strategy to ensure HBV infection. Ellagic acid intake resulted in reduced HBeAg levels, recovery of T/B cell response, IgG antibody production, cytokine release, and cytotoxic T-lymphocyte response, demonstrating that it should be used as a medicinal agent for HBV carriers (Kang et al. 2006). Thanks to its immunomodulatory and antioxidant properties, Ellagic acid also protects against IV infection when combined with oseltamivir and isoprinoside (Pavlova et al. 2018). Finally, **phyllaemblicin B**, the primary ellagitannin compound found in *Phyllanthus emblica* L., demonstrated an antiviral effect against myocarditis induced by CVB3 by decreasing the viral titer, apoptosis, and inflammation both in vivo (12 mg/kg/day, intravenous administration) and in vitro (IC_50_ = 7.75 ± 0.15 μg/mL) (Wang et al. 2009).

### Alkaloids

Alkaloids are molecules of natural origin containing hydrogen, carbon, oxygen, and nitrogen and generally exist as small nitrogenous compounds in about 20% of plant species. These natural active metabolites have been demonstrated to interact whit several targets making them candidates for exerting antiviral activity. Alkaloids have indeed shown to possess antioxidant and anti-inflammatory activity and the ability to inhibit DNA and RNA synthesis, determining a viral replication blockage. **Epigoitrin**, a natural alkaloid isolated from *Isatis tinctoria* L. (Brassicaceae), protected against IAV infection in investigations employing in vitro and in vivo stress-induced models (88 or 176 mg/kg/day, orally administered to infected mice)*.* Differently from the natural active molecules seen so far, epigoitrin reduced IV-susceptibility via mitochondrial antiviral signaling since it might reduce mitofusin-2 (MFN2) expression determining an increase in the mitochondria antiviral signaling expression and then an enhancement of IFN-*β* and interferon-inducible transmembrane 3 (IFITM3) production. Therefore, epigoitrin acted by maintaining the antiviral signaling of mitochondria antiviral signaling, generally suppressed under restraint stress by MFN2, ensuring INF-*β* release and so reducing viral replication (Luo et al. 2019). Moreover, it was seen that epigoitrin existed as a stereometric mixture of an equal amount of epigoitrin of (*R*)–goitrin, and goitrin or (*S*)–goitrin, where the *S* enantiomer claimed a greater activity than the *R*-enantiomer (Nie et al. 2020). Likewise, **indirubin**, a bis indole alkaloid from *Isatis tinctoria* L. (Brassicaceae), significantly restored MAVS expression and promoted IRF3 phosphorylation, thereby increasing IFN-*β* production both in vitro and in vivo (2.5 or 5 mg/kg/day, orally administered to infected mice). Type I IFNs are involved in stimulating the expression of ISGs genes which play an essential role in contrasting viral infection; an example of ISG is IFITM3, involved in restricting IAV replication by blocking the fusion between virus and host cell membrane. Indirubicin was demonstrated to improve not only IFN-*β* by MAVS activation but also IFITM3 protein expression, which normally decreases during IAV infection. Furthermore, this alkaloid down-regulated the expression of pro-inflammatory cytokines (TNF-*α*, IL-1*β*, and IL-6) and up-regulated the production of anti-inflammatory mediators like IL-10 leading to the reduction of IAV-induced inflammation. Thus, indirubin exerted its anti-IAV activity by decreasing the susceptibility to IAV virus and pro-inflammatory cytokines levels and maintaining the mitochondria function and morphology, ensuring IFN-*β* production controlled by mitochondria antiviral signaling (Jie et al. 2017). Indirubin also showed a virucidal effect against JEV infection, even if the effective mechanism of action needs to be understood (Chang et al. 2012). Protection against IAV infection was also evidenced for **homonojirimycin**, from *Commelina communis* L. (Commelinaceae), which was found to protect mice (1 mg/kg two times daily, orally administered) from infection and have anti-inflammatory activity by inhibiting IL-6 and TNF-*α* and up-regulating IFN-*γ* and IL-10 expression (Zhang et al. 2013). Similarly, reduced IAV-induced inflammation and improved pulmonary inflammation and histopathological changes were seen for **oxymatrine,** a quinolizidine alkaloid obtained from *Sophora* (Fabaceae) genus. The oxymatrine administration to IAV-infected mice (60 or 120 mg/kg/day by oral gavage) suppressed IAV-induced activations of TLR4, p38 MAPK, and NF-*κ*B pathways (Dai et al. 2018a). Likewise, oxymatrine inhibited viral replication and regulated the release of cytokine involved in the host defence mechanism like IFN-γ, leading to a lowered inflammation in the heart and protection of mice from CVB3-induced myocarditis (3.125, 6.25, 12.5, and 25 mg/kg/day, intraperitoneal injection) (Jiang et al. 2017). However, the most important antiviral activity claimed for oxymatrine is against HBV since it is clinically used to treat chronic viral hepatitis B in China (Chen et al. 2001). In vitro and in vivo investigation demonstrated that oxymatrine inhibited HBV DNA replication and determined a reduction of HbsAg and HbeAg by stimulating innate immunity to increase the production and release of IFN-γ in CD4^+^T cells (Chen et al. 2001; Lu et al. 2004; Sang et al. 2017). Specifically, treatment with oxymatrine (200 mg/kg/day, intraperitoneal injection) triggered a modification of the immune defence against hepatitis B infection through an up-regulation of Th1 cytokines (IFN-γ and IL-6) and downregulation of Th2 cytokines (IL-4 and IL-10), which determined an improvement in HBV inhibitory activity (Dong et al. 2002). The anti-HBV of oxymatrine was also demonstrated in clinical studies since this alkaloid abrogated HBV DNA and HbAg levels by down-regulating HBV-specific cytotoxic T lymphocyte (CTL) surface programmed death receptor-1 (PD-1) expression and increasing HBV-specific CTL levels (Gu et al. 2012; Lu et al. 2003). These effects resulted in a reduction of hepatic fibrosis and inflammation, as evidenced in a randomized, double-blind, placebo-controlled, multicenter clinical study (capsules of 300 mg, 3 times a day), where a decreased level of hepatic fibrosis serum markers, such as hyaluronic acid and type III procollagenic peptide (P III P), was seen (Mao et al. 2004). Moreover, oxymatrine was effective in reducing HBV DNA and HBeAg levels in chronic hepatitis B patients (0.2 g/day) resistant to lamivudine and, if used in combination with lamivudine (lamivudine 100 mg/day + oxymatrine 0.2 g/day), reduced the incidence of lamivudine resistance after one year of treatment (Wang et al. 2011). The modulation of innate immunity also characterized the anti-EV71 activity. It was indeed demonstrated that oxymatrine and five other quinazoline alkaloids, **matrine**, **sophoramine**, **sophocarpine, sophoridin**e, and **oxysophocarpine** inhibited EV71 replication by increasing the levels of T cells, such as CD3 + , CD4+ , and CD8 + (Yang et al. 2015, 2012b). Among them, oxysophocarpine exerted the greatest activity. In fact, its administration (7.5, 15, and 30 mg/kg) exhibited an increase in the survival time of infected mice, and, at a dose of 15 mg/kg/day, also inhibited the virus replication in mice muscles, suggesting the possible employment of oxysophocarpine to control fatal EV71 outbreaks (Yang et al. 2015). **Harmine**, a *β*-carboline alkaloid isolated from *Peganum harmala* L. (Nitrariaceae), is another alkaloid with potential anti EV71 activity. The mechanism of action by which this compound acted is not well documented, but it seemed that it might repress EV71 replication in mice (12.5 μg/mL/day, intraperitoneal injection) by inhibiting the NF-*κ*B pathway leading to a reduction of oxidative stress (Chen et al. 2018). Known is instead the mechanism for the anti-HSV infection of harmine and **harmaline** (Chen et al. 2018), a *β*-carboline alkaloid extracted from *Ophiorrhiza nicobarica* N.P.Balakr. (Rubiaceae). Specifically, it was seen that harmaline interfered with the HSV immediate-early transcriptional events by avoiding the recruitment of lysine-specific demethylase-1 from its immediate-early promoter during immediate-early complex transcription. This complex is a decisive factor for HSV latency or lytic cycle, making harmaline a molecule capable of preventing the multiplication and reactivation of the HSV (Bag et al. 2014, 2013). An anti-HSV activity was also evidenced for the morphinan alkaloid **6,7-di-*O*-acetylsinococuline** obtained from the root of *Stephania cepharantha* Diels (Menispermaceae), the diterpene alkaloid **benzoylmesaconine** from *Aconitum* ssp. tuber (Ranunculaceae), and the bisbenzylisoquinoline alkaloid **tetrandrine** from *Stephania tetrandra* S. Moore (Menispermaceae). 6,7-Di-*O*-acetylsinococuline demonstrated a promising anti-HSV activity using in vivo model of infected mice (10 and 25 mg/kg/day, orally administered); however, a narrow therapeutic index was seen (Nawawi et al. 2001). In contrast, benzoylmesaconine acted similarly to oxymatrine by modulating the host immune defence system. Benzoylmesaconine (1 μg/kg, 2 days before and 1 and 3 days after infection, orally administered), indeed, through the induction of antagonistic CD4+ T cells, might improve the resistance to the virus in HSV-1 infected mice (Kobayashi et al. 1998). On the other hand, tetrandrine (15 mg/kg, administered parenterally twice daily from day 7 after infection) exerted an anti-viral and anti-inflammatory activity by reducing mRNA expression of IL-6 and inhibiting IL-1*β* and TNF-*α* release, thereby blocking HSV viral replication in infected mice (Hu et al. 1999).

As well as oxymatrine, another alkaloid with an anti-viral activity against different virus strains is **berberine**, an isoquinoline alkaloid isolated from plants of *Berberis* genus (Berberidaceae) which displayed to be effective on HBV, CHIKV, CVB3, and IAV infection (Dai et al. 2021; Subaiea et al. 2017; Varghese et al. 2016; Wu et al. 2011c; Yan et al. 2018). In all cases, the reduction in viral replication was accompanied by a decrease in the inflammatory markers. For instance, berberine significantly reduced viral load and CHIKV-induced inflammatory diseases in mouse models (10 mg/kg/day, administrated intraperitoneally) by blocking ERK, p38, and JNK (Varghese et al. 2016) and showed a strong reduction of IAV growth (0.1 g/kg/day or 20 mg/kg/day, intraperitoneally and orally administrated, respectively), as well as suppression of the expression of TLR7/NF-*κ*B signaling molecules and inhibition of the initiation of virus-induced T-cell responses and release of pro-inflammatory cytokines in the lungs (Wu et al. 2011c; Yan et al. 2018). **Lycorine**, an alkaloid from *Lycoris radiate* (L’Hér.) Herb. (Amaryllidaceae), was also reported to display antiviral activity in vitro and in vivo in different virus strains like EV71, CoVs, and Zika (Chen et al. 2020; Liu et al. 2011; Shen et al. 2019). A viral replication reduction was seen in all these cases, but only for Zika the mechanism of action was discovered. Specifically, in vivo investigation on infected mice showed that lycorine (1, 5, and 10 mg/kg/day, intragastric administration) blocked Zika replication, acting on multiple targets since it possessed the ability to bind HSP70 and Zika NS3 and NS5 protein (Chen et al. 2020). With the same anti-viral mechanism as lycorine, another Amaryllidaceae alkaloid, **pancratistatin**, probably acted since it was demonstrated to reduce the RNA replication of different flaviviruses like JEV (IC_50_ = 0.022 µg/mL), yellow fever (IC_50_ = 0.016 µg/mL), and DENV 4 (IC_50_ = 0.063 µg/mL) (Gabrielsen et al. 1992). Specifically, pancratistanin (4 and 6 mg/kg/day, administered intraperitoneally) and its analogous, **7-deoxypancratistatin** (40 mg/kg/day, administrated subcutaneously), increased the survival rate of JEF-infected mice (Gabrielsen et al. 1992). **Tetrahydropalmatine**, the main compound reported in genera of *Stephania* and *Corydalis,* was also in vivo (2 mg/kg, administered intraperitoneally twice a day) demonstrated to reduce JEV replication and exert neuroprotective effects by reducing the levels of TNF-α, IFN-γ, MCP-1, and IL-6 (Lixia et al. 2018). **Castanospermine**, a polyhydroxyalkaloid derived from the seeds of the *Castanospermum australe* A. Cunn. & C.Fraser (Fabaceae), demonstrated to be broadly active in vitro against many viruses by inhibiting α-glucosidase I and II, an enzyme that plays an important role in viral maturation (Schlesinger et al. 1985; Sunkara et al. 1990; Whitby et al. 2005; Yamashita et al. 1996; Whitby et al. 2005). Through this mechanism of action, castanospermine probably reduced the production and infectivity of DENV particles and prevented mortality in infected mice model (10, 50, and 250 mg/kg/day, administered intraperitoneally) (Whitby et al. 2005). However, this alkaloid is characterized by low absorption and bioavailability; for this reason, by integrating a lipophilic butanoyl side-chain, its oral prodrug celgosivir, with the same anti-viral activity, was developed (Durantel 2009; Rathore et al. 2011; Watanabe et al. 2012).

Other alkaloids exerted antiviral activity against rabies virus, human syncytial virus (hRSV), CVB3, and COVID-19. **Bufotenine**, a tryptamine isolated from plants of Mimosaceae family, exerted antiviral activity against Rabies virus infection in vitro* and *in vivo by inhibiting the penetration of rabies virus into the host cells through the inhibition of the nicotinic acetylcholine receptor with an apparent competitive mechanism (Vigerelli et al. 2014). However, a more recent in vivo study questioned this mechanism of action as bufotenine did not appear to interfere with the acetylcholine response in skeletal muscle, indicating that its mechanism of action does not block virus entry due to nAChR antagonism. Furthermore, it was observed that bufotenine did not passively penetrate cell membranes, indicating the need for complementary structures, like cell receptors, for cell penetration (Vigerelli et al. 2020). This mechanism seems to underlie the increase in the survival rate of intrathecally Rabies virus-infected mice after bufotenine administration (0.63, 1.05, and 2.1 mg/animal per day) (Vigerelli et al. 2018, 2020). **Cyclopamine**, a steroidal alkaloid from *Veratrum ssp.* (Melanthiaceae), demonstrated to inhibit hRSV infection by inhibiting the Smoothened receptor (Smo) in a BALB/c mouse model of infection (30 and 100 mg/kg intraperitoneally). Specifically, it impaired the hRSV RNA-dependent RNA polymerase complex by reducing the viral anti-termination factor M2-1expression levels. This mechanism was confirmed by the fact that a single R151K mutation in M2-1 conferred viral resistance to cyclopamine (Bailly et al. 2016). **Sophoridine**, an alkaloid extracted from *Sophora flavescens* Aiton (Fabaceae), was widely studied for its beneficial and therapeutic effect on cardiac function, including in acute and chronic viral myocarditis caused by CVB3. This alkaloid demonstrated, through in vivo experimentation on infected mice models (20 and 40 mg/kg/day, orally administrated), an anti-CVB3 activity by regulating cytokine expression since sophoridine significantly down-regulated the mRNA expression of TNF-α and up-regulated IL-10 and IFN-γ mRNA expression (Zhang et al. 2006b). **Emetine**, an isoquinoline alkaloid, reported potent in vitro (EC_50_ = 0.147 nM) activity against SARS-CoV-2 and *in ovo* antiviral efficacy against infectious bronchitis virus (EC_50_ = 2.3 ng/egg) (Kumar et al. 2021). Furthermore, a preliminary study on patients with COVID-19 infection showed that low-dose emetine (3.6 mg via oral administration 3 times per day for 10 days) in combination with conventional antiviral drugs improved clinical symptoms without apparent adverse effects (Fan et al. 2021).

### Diarylheptanoids

Diarylheptanoids frequently occur in the rhizomes of many plants and are structurally characterized by two aromatic rings linked by a chain with seven carbons. In *Alpinia officinarum* Hance (Zingiberaceae), diarylheptanoids are considered the main bioactive compounds, with several pharmacological activities, including antiviral (Abubakar et al. 2018). Among them, **7-(4′′-hydroxy-3′′-methoxyphenyl)-1-phenyl-4*E*-hepten-3-one** and **(5*S*)-5-hydroxy-7-(4-hydroxyphenyl)-1-phenylhept-3-one** has been reported to exhibit anti-IV activity in vitro by suppressing the expression of viral mRNA antigens while no effect was seen for adsorption or invasion of host cells. (5*S*)-5-Hydroxy-7-(4-hydroxyphenyl)-1-phenylhept-3-one was more effective in contrasting viral infection in vitro than 7-(4′′-hydroxy-3′′-methoxyphenyl)-1-phenyl-4*E*-hepten-3-one. However, this last was more active in vivo (30 and 100 mg/kg via oral administration 3 times daily) since no effect was found for (5*S*)-5-hydroxy-7-(4-hydroxyphenyl)-1-phenylhept-3-one, and this was probably due to the differences in the structure, indicating that the methoxy group linked to the aromatic ring of 7-(4′′-hydroxy-3′′-methoxyphenyl)-1-phenyl-4E-hepten-3-one was favourable in contrasting the influenza virus in vivo (Sawamura et al. 2010). In contrast, (5*S*)-5-hydroxy-7-(4-hydroxyphenyl)-1-phenylhept-3-one demonstrated RSV antiviral activity in vivo (30 mg/kg, orally 3 daily time administration) and in vitro (EC_50_ = 40.7 ± 3.5 µg/mL), and, also in this case, the importance of chemical structure was highlighted. In particular, the hydroxy group seemed to be essential in reducing virus titer than other diarylheptanoids containing a methoxy group in the carbon chain (Konno et al. 2013). Another diarylheptanoid **curcumin**, a bright yellow-colored bioactive diarylheptanoid from Zingiberaceae plants, showed remarkable antiviral activity against several viruses. In this respect, curcumin has proven its efficacy against HSV-1 (Zandi et al. 2010), IV es PR8, H1N1, and H6N1 (Chen et al. 2011), coxsackievirus (Si et al. 2007), and others. Specifically, curcumin might inhibit viral entry and replication by inhibiting either HA or NA, thanks to the presence of the hydroxyl group in the benzene-ring meta-position and the central carbon-chain double bonds (Lai et al. 2020). Further, an in vivo investigation on infected mice demonstrated that curcumin (50 and 150 mg/kg/day, administered by oral gavage) avoided IAV-induced oxidative stress and inflammation by activating the Nrf2-HO-1 pathway and inhibiting TLR2/4, p38/JNK MAPK, and NF-κB signaling pathway leading to the increase of INF-β and the suppression of IAV replication (Dai et al. 2018b). This mechanism also underlies the amelioration of IVA-induced myocarditis in mice treated with curcumin (100 mg/kg/day, given orally) (Liu et al. 2013). These anti-inflammatory and antioxidant activities are also related to the curcumin's anti-viral action against HCNV infection since an up-regulation of SOD and GSH levels and a down-regulation of TNF-α and IL-6 were observed in infected mice (12.5, 25, and 50 mg/kg, intragastrical administration) (Lv et al. 2014b).

### Benzoic acids

Benzoic acids are organic compounds consisting of a benzene ring with at least one carboxyl group. Among these compounds, **ginkgolic acid**, extracted from the leaves and seed coats of *Ginkgo biloba* L., has demonstrated broad antiviral activity. In particular, this compound inhibited HSV and the HSV- ACV^R^ by preventing the initial fusion event through the block of either fusion or viral protein synthesis. This mechanism resulted in the improvement of cutaneous infection in HSV-infected mice after topical treatment with ginkgolic acid (10 mM ginkgolic acid in 2.5% hydroxyethylcellulose gel) (Bhutta et al. 2021). Another promising benzoic acid with antiviral activity is **protocatechuic aldehyde**, or 3,4-dihydroxybenzaldehyde, a naturally-occurring phenolic aldehyde isolated from *Salvia miltiorrhiza* Bunge, which was demonstrated to inhibit HBV DNA replication and HBsAg and HBeAg secretion both in vivo (25, 50, and 100 mg/kg, intraperitoneally, twice daily) and in vitro (EC_50_ = 3.94 ± 1.52 and 2.46 ± 0.38 μg/mL for avoiding HBsAg and HBeAg production, respectively) probably by inhibiting HBV polymerase activity (Zhou et al. 2007).

### Coumarins

Coumarins are compounds isolated from different plant families and are structurally formed by a benzene moiety fused to an α-pyrene ring known as benzopyrene. **Isofraxidin**, an anticoagulant compound extracted from the plants *Sarcandra glabra* Thunb. (Chloranthaceae) and *Eleutherococcus senticosus* (Rupr. et Maxim.) Maxim*.* (Araliaceae), exhibited therapeutic effects on IAV-induced severe pulmonary infection in vitro and in vivo*.* This coumarin did not inhibit IAV replication but significantly reduced lung IAV-induced inflammation, suppressed platelet aggregation through PI3K/AKT and MAPK pathways regulation, and decreased the serum levels of IL-1*β*, IL-6, TNF-*α*, and MIP-2 in infected mice (10, 20, and 40 mg/kg/day, orally administrated) (Jin et al. 2020). Contrarily, **calanolide A**, a pyranocoumarin found in several species belonging to *Calophyllum* genus, directly avoided HIV replication by inhibiting HIV-1 reverse transcriptase both in vivo and in vitro (Currens et al. 1996; Xu et al. 1999). Another coumarin-like compound, **dicoumarol**, expressed a high antiviral activity on HBV infection by inhibiting the NQO1 and then avoiding HBx expression and recruitment to cccDNA. In this way, cccDNA transcriptional activity was inhibited, thereby preventing HBV RNAs, HBV DNA, HBsAg, and HBc protein synthesis in either HBV-infected cells or humanized liver mouse models (10 and 20 mg/kg/day, intraperitoneally administrated) (Cheng et al. 2021).

### Other natural compounds

The limonoid **toosendamin**, from *Melia azedarach* L. (Meliaceae), demonstrated an anti-IAV activity both in vivo (1 mg/kg/day, orally administrated) and in vitro by inhibiting the infection early-stage through binding the polymerase acidic (PA) protein of the IAV polymerase complex with a greater extent than the licensed PA protein inhibitor. In this way, toosendamin did not affect HA or NA directly but inhibited the mRNA synthesis of HA, nucleoprotein (NP), and M2, and the expression of NP, PA, M2, and NS1, leading to the suppression of IAV infection (Jin et al. 2019). **Resveratrol** is a natural polyphenol stilbene mainly found in fermented grapes, mulberry, red wine, and peanuts. It inhibited a wide variety of pathogenic human and animal viruses, and sometimes it interfered with infection by altering cellular pathways rather than acting directly against the virus itself. In this respect, different papers reported that the resveratrol activity relies on its implication with the immune system and with the innate immunity factors, particularly IFN-α, IFN-γ, TNF-α, and IL-12, whose levels are considered critical for the antiviral activity. Resveratrol was shown to be an inhibitor agent of varicella-zoster virus (Docherty et al. 2006), HCMV (Evers et al. 2004), and IVes (Palamara et al. 2005). Specifically, resveratrol strongly inhibited IAV influenza infection by blocking the viral ribonucleoprotein nuclear cytoplasmatic translocation and reducing the late viral protein expression, probably by inhibiting protein kinase C activity and its signaling pathway. This mechanism underlying the improved survival rate and decreased lungs viral titer observed in infected mice after resveratrol administration (1 mg/kg/day via intraperitoneal injection) (Palamara et al. 2005). The resveratrol anti-inflammatory properties were instead related to the anti-RSV activity since its administration to mice (30 mg/kg/day, intraperitoneal administration) reduced viral titers and airway inflammation (Zang et al. 2015, 2011). These effects were related to the regulation of Toll-like receptor 3 (TLR3) and M2 expression, the TRIF signaling pathway inhibition, and the reduction of nerve growth factor (NGF) levels, thereby lowering IFN-γ and IL-6 levels related to RSV-induced airway inflammation (Xie et al. 2012; Zang et al. 2015, 2011). Resveratrol also avoided the in vitro replication of HSV-1 and HSV-2 in a dose-dependent manner by decreasing the virus yield through inhibition of an early phase of the replication cycle and reduction of early viral protein production (Cheng et al. 2014). This activity resulted in the improvement of RSV infection in mice after resveratrol topical application (Docherty et al. 2005, 2004).

**Hypericin** and **pseudohypericin** are two aromatic polycyclic diones, found in many *Hypericum* species (Hypericaceae) and isolated for the first time from *Hypericum perforatum* L. In general, both compounds exhibit a broad spectrum of antiviral activity against several viruses (Karioti and Bilia 2010). Tang et al. (1990) demonstrated the virucidal effect of hypericin against enveloped viruses (Mo-MuLV, herpes simplex, IV A), when the virus was incubated with hypericin before infection. This effect was also demonstrated i*n vivo* since hypericin and pseudohypericin (50 µg/mL), if pre-incubated with the virus for 1 h at 37 °C, were highly effective in preventing HSV-1-induced encephalitis and death in CD-1 mice, displaying 100% survival six days after infection respect to the control, while they were only moderately effective if pre-incubated with the virus for 1 h at 4 °C, and not effective if administered concurrently with the virus. These data suggest that the utility of hypericin and pseudohypericin could depend on pre-treating cells with these compounds to create an antiviral state (Tang et al. 1990). The carotenoid **crocetin**, from *Crocus sativis* L., demonstrated strong antiviral activity against CVB3-induced myocarditis. It was indeed seen that crocetin (2.5 and 5 mg/kg/day, intraperitoneally administrated) reduced IL-17 and ROCK2 expression, thereby exerting a cardioprotective effect in infected mice by decreasing viral replication and inflammatory response (Qin et al. 2021). Finally, the *seco*-pregnane steroidal glycoside **paniculatumoside C**, isolated from *Cynanchum paniculatum* (Bunge) Kitag. Ex H.Hara (Apocynaceae)*,* has revealed a significant antiviral property by inhibiting viral replication of several RNA viruses, including the Getah virus, eastern equine encephalitis virus, tobacco mosaic virus, and sindbis virus (Li et al. 2007). Specifically, in vivo investigation showed that paniculatumoside C administration (5, 50, and 100 mg/kg/day) protected mice from lethal sindbis virus infection. Using the tobacco mosaic virus and sindbis virus as models, investigations about the action mechanism suggested that this compound predominantly acted by suppressing the viral subgenomic RNA(s) expression without interfering with viral genomic RNA accumulation (Li et al. 2007).

## Conclusions

In recent years, particular attention has been paid to the pandemic threat of viruses that can have harmful effects on humans, plants, and animals. Viruses have started the most lethal and terrifying illnesses the world has ever known; an example is the ongoing 2019 coronavirus pandemic (COVID-19). Vaccination is the main strategy used to prevent viral infection; however, its long-term efficacy might be compromised due to viral mutation. On the other hand, the major issue in viral infection is the rapid development of resistance against licensed antiviral drugs making the need to identify new antiviral agents characterized by the highest efficacy and less toxicity. Several traditional medicinal plants have been reported to possess strong antiviral activity thanks to specialized metabolites able to act through multiple mechanisms, distinguishing them from the usual synthetic drugs that mainly act on a single target. In this systematic review, the main antiviral mechanisms of action of natural molecules were described making them promising antiviral agents. We have resumed in a systematic way all the knowledge that is available on plant natural products tested in vivo up to now. It is evident, as natural products that have been mostly tested in vivo on animal models have not always demonstrated an outstanding antiviral effect, at least on the tested virus strain, while others, belonging to different classes, have shown promising results acting on both inflammation and/or on virus replication. On the other hand, it is possible to observe that just a few of the most promising molecules have been tested in clinical studies. In fact, only limited clinical investigations were successfully done proving an interesting and transferable use at human level as drugs; (e.g., silibinin or oxymatrine on hepatitis). This lack of studies is mainly due to the not easy patentability and profitable uses of the plant small molecules chemical structure since they are common knowledge. Unfortunately, to obtain a patent of a molecule an enormous effort in terms of drug research and development and of money (hundreds of millions of dollars) are necessary. The recovery of the expenses for the pharmaceutical companies, mainly involved in this process, by either licensing the patent to other manufacturers or by acting as the sole manufacturer themselves is not often possible; so due to the clinical studies costs and also to the presence of other pharmacological strategies, such as synthetic antivirals or vaccines, the plant small molecules listed in this review have not obtained the full attention they merit. However, apart from this issue, there are others upstream since several challenges arise during drug discovery or natural compounds in vivo studies, e.g. solubility, bioavailability, and toxicity that may occur after plant natural compounds bioactivation. One of the critical points in many plant specialized metabolites is their biotransformation and bioavailability in human. The biotransformation of many classes of these compounds has been a controversial issue over the last decades. Moreover, high polar molecules, overall tannins, flavonoids, and their glycosides, could be adsorbed and biotransformed by intestinal microbiota; moreover several derivatisations have been shown to be effective in increasing solubility and reducing toxicity (Owen et al. 2022). Regarding bioactivation, it was seen that several herb-induced toxicities are a consequence of herbal constituents' biotransformation into electrophilic reactive metabolites that may covalently link vital macromolecules into the body. For example, at physiologically relevant concentrations, furanocoumarins may be bioactivated in molecules able to inactivate enzymes involved in drug metabolism leading to the manifestation of herb-drug interaction. In the same way, the bioactivation of several terpenoids may lead to toxic compounds; hence it is important to focus research on the potential toxicity of natural compounds and their bioactivation products (Wen et al. 2019). Finally, it is important not to label a molecule of natural origin as biologically active just basing on in vitro assay using cell models since some compounds may be fluorescent or strongly coloured, thereby giving positive signals and so false readings even if, for example, in the case of an interaction with an enzyme, a protein is not present. Moreover, several plant small molecules, mainly belonging to the polyphenol group, were reported to interact with different and numerous protein targets and to present panacea-like properties. This behaviour over the years brings to the description of a wrong mechanism of action for several plant compounds due to their nonspecific and promiscuous activity. It’s largely reported that this characteristic is due to chemical (photo)reactivity, redox cycling, metal chelation, interferences with the assay technology, membrane disruption, etc., which depends on the presence of several substructures in the molecule defined as interfering. An acronym used to classify these kinds of molecules is PAINS (pan-assay interference compound) and several new guidelines were published to help researchers to avoid money and time consumption in the study of their bioactivity (Baell and Walters 2014; Bisson et al. 2016). Actually, the possible PAINS can be discovered experimentally or by using prognostic values to highlight possible aggregation and/or interference based only on the chemical structure of the tested compound. It’s almost clear that PAINS are a real issue and a preliminary non-critical analysis should be avoided before going on with research projects involving these compounds. On the other hand, other reports indicated that a molecule classified as PAINS does not have high assay promiscuity, therefore also this concept should not be blindly used but verified using data coming for example from different and orthogonal assays (Aldrich et al. 2017). From our literature survey, the antiviral in vivo studies on plant small molecules stated some benefits from their use, so it will be crucial to design different kinds of clinical trials to provide conclusive evidence of their efficacy. Moreover, compounds emerging from preliminary antiviral in vivo assays could be an important starting point as chemical scaffolds to develop by medicinal chemistry approaches new molecules with antiviral activity. Therefore, scientific research on antiviral plant natural products should be carried out with critical issues covering all the previous aspects. Moreover, for clinical trials is important to give preliminary evidence of specialized plant compounds' antiviral efficacy; for this reason, this review mainly focuses on in vivo investigation and may provide the scientific basis for future studies on the applicability of natural active metabolites for preventing and treating viral infection.
